# Evaluation of Commercial Diagnostic Assays for the Specific Detection of Avian Influenza A (H7N9) Virus RNA Using a Quality-Control Panel and Clinical Specimens in China

**DOI:** 10.1371/journal.pone.0137862

**Published:** 2015-09-11

**Authors:** Dawei Shi, Shu Shen, Xingliang Fan, Suhong Chen, Dayan Wang, Changgui Li, Xing Wu, Lili Li, Dongting Bai, Chuntao Zhang, Junzhi Wang

**Affiliations:** 1 Key Laboratory of the Ministry of Health for Research on Quality and Standardization of Biotech Products, National Institutes for Food and Drug Control, Beijing, People’s Republic of China; 2 WHO Collaborating Center for Standardization and Evaluation of Biologicals, Beijing, People’s Republic of China; 3 Beijing Institute of Radiation Medicine, Beijing, People’s Republic of China; 4 Chinese National Influenza Center, National Institute for Viral Disease Control and Prevention, Chinese Center for Disease Control and Prevention, Beijing, People’s Republic of China; 5 WHO Collaborating Center for Reference and Research on Influenza, Beijing, People’s Republic of China; National Institute for Viral Disease Control and Prevention, CDC, China, CHINA

## Abstract

A novel avian influenza A H7N9-subtype virus emerged in China in 2013 and threatened global public health. Commercial kits that specifically detect avian influenza A (H7N9) virus RNA are urgently required to prepare for the emergence and potential pandemic of this novel influenza virus. The safety and effectiveness of three commercial molecular diagnostic assays were evaluated using a quality-control panel and clinical specimens collected from over 90 patients with confirmed avian influenza A (H7N9) virus infections. The analytical performance evaluation showed that diverse influenza H7N9 viruses can be detected with high within- and between-lot reproducibility and without cross-reactivity to other influenza viruses (H1N1 pdm09, seasonal H1N1, H3N2, H5N1 and influenza B). The detection limit of all the commercial assays was 2.83 Log_10_ copies/μl [0.7 Log_10_TCID_50_/mL of avian influenza A (H7N9) virus strain A/Zhejiang/DTID-ZJU01/2013], which is comparable to the method recommended by the World Health Organization (WHO). In addition, using a WHO-Chinese National Influenza Center (CNIC) method as a reference for clinical evaluation, positive agreement of more than 98% was determined for all of the commercial kits, while negative agreement of more than 99% was observed. In conclusion, our findings provide comprehensive evidence for the high performance of three commercial diagnostic assays and suggest the application of these assays as rapid and effective diagnostic tools for avian influenza A (H7N9) virus in the routine clinical practice of medical laboratories.

## Introduction

Since February 2013, confirmed cases of human infection by a novel avian influenza A H7N9-subtype virus have been continuously identified in China. As of November 16, 2014, a total of 457 confirmed cases had been reported, including 177 deaths [1]. This is the first time that avian influenza A H7N9-subtype virus infection has been reported in humans [2]. The virus has been identified as a novel reassortant influenza virus that differs genetically from the other previously identified avian influenza A H7N9-subtype viruses. It carries six internal genes originating from the avian H9N2 influenza viruses but has the hemagglutinin (HA) and neuraminidase (NA) genes from the avian H7 and N9 influenza viruses, respectively [3]. Investigations of viral sequences revealed that this virus contains several mammalian-adaptive mutations that are known to be associated with improved invasion and replication of avian influenza viruses in mammals [2, 3]. Subsequent experimental studies demonstrated that this virus could replicate in the respiratory tracts of various animals, including non-human primates, and could be transmitted by direct contact and aerosolization in the ferret [4–6]. In addition, it was demonstrated that infected chickens could survive and shed virus for up to 14 days without any obvious clinical signs of the disease [5]. The observed low pathogenicity of avian influenza A (H7N9) virus in poultry weakens the warning effects of symptom-based screening for infected poultry, thus facilitating the spread of this virus among poultry and increasing the risk of human exposure. Although no sustained human-to-human transmission has been determined, several cases of family clusters have been identified in some provinces of China that experienced the outbreak, suggesting that limited non-sustained human-to-human transmission may occur under some circumstances, such as long-term, unprotected close contact [7, 8]. Considering the probable lack of pre-existing immunity among humans to this newly emerged H7N9 virus, this virus poses a great threat to national and global public health.

Laboratory-designed, in-house nucleotide detection assays have been developed and used in the public health laboratories of the Chinese National Influenza-Like Illness Surveillance Network (CNISN). These assays are currently the main tools employed for the rapid identification of avian influenza A (H7N9) virus and have played a critical role in early responses to the outbreak of H7N9 avian virus infection [9–15]. However, because the possibility of sustained human-to-human transmission cannot be completely excluded and because new cases have continuously accumulated, appearing as a second epidemic wave in winter 2013 and spring 2014 in China [1, 16], careful and persistent monitoring of avian influenza A (H7N9) virus is necessary for urgent and long-term responses to threats from the virus. Thus, enhanced detection capacities with sustained quality-control measures are urgently needed to allow a better response to the current outbreak of this novel influenza virus and to improve preparedness for its re-emergence or even a potential pandemic in the future. Compared with in-house assays, commercial diagnostic kits typically provide a more sustainable alternative source of accurate detection tests, as they support larger-scale production, certified manufacturing practices, well-studied product performance and stable quality control; hence, they can be used in a broad range of clinical laboratories.

Therefore, to meet the increasing need for detection, the China Food and Drug Administration (CFDA) has approved three commercial diagnostic products for specifically detecting avian influenza A (H7N9) virus RNA, which can be used in the laboratories that are not a part of CNISN under the Emergency Use Authorization (EUA) [17]. Here, to ensure the safety and effectiveness of these commercial molecular diagnostic assays, we conducted analytical and clinical evaluations using a well-characterized quality-control panel of viral cultures and a sufficient number of clinical specimens collected throughout the major epidemic regions of China.

## Materials and Methods

### Ethics Statement

The study protocol and informed consent documents were reviewed and approved by the Ethics Committee of the First Affiliated Hospital, College of Medicine, Zhejiang University (ZJU Hospital), the Shanghai Public Clinical Health Center (SPHCC), the Nanjing Municipal Centers for Disease Control and Prevention (Nanjing CDC), the Zhejiang Provincial Centers for Disease Control and Prevention (Zhejiang CDC), the Guangdong Provincial Centers for Disease Control and Prevention (Guangdong CDC), the Guangzhou Municipal Centers for Disease Control and Prevention (Guangzhou CDC) and the Jiangsu Provincial Centers for Disease Control and Prevention (Jiangsu CDC). Written informed consent for the research use of clinical samples was obtained from all of the patients involved in the study.

### Establishment of the quality-control panel

To analytically evaluate the commercial assays, a panel of quality-control materials needs to be established. The processes of the panel’s establishment include the following sequential steps: virus isolation and culture, sequence confirmation of viral cultures, diluting, dispensing and freeze-drying the viral cultures, quantification and stability evaluation of the quality-control panel.

#### Virus isolation and culture

Five strains of avian influenza A (H7N9) virus (H7N9 virus) were isolated and cultured in the BSL-3 laboratory to establish the quality-control panel. Briefly, throat-swab specimens obtained from patients were maintained in viral-transport medium, and H7N9 virus strains were obtained by propagating the clinical specimens in the allantoic sacs and amniotic cavities of 9-to-11-day-old specific pathogen-free embryonated chicken eggs for 48 to 72 h at 35°C. The virus titer (TCID_50_ or HA titer) was determined according to the protocols recommended by the WHO [18, 19]. The culture supernatants of two H7N9 virus strains (A/Zhejiang/DTID-ZJU01/2013 and A/Zhejiang/DTID-ZJU02/2013) were prepared by the State Key Laboratory for Diagnosis and Treatment of Infectious Diseases, College of Medicine, Zhejiang University, China, and the viral titers were 10^5.7^ and 10^4.6^ TCID_50_/mL, respectively. The other three H7N9 strains were cultured by the National Institute for Viral Disease Control and Prevention (part of the Chinese Center for Disease Control and Prevention), which is a WHO Collaborating Center for Reference and Research on Influenza (WHO CC). The titers of the viruses were 128 HA units (A/Shanghai/1/2013), 64 HA units (A/Shanghai/2/2013) and 64 HA units/10^7.2^ TCID_50_/mL (A/Anhui/1/2013). The viral cultures of five H7N9 viruses were inactivated with beta-propriolactone, and their inactivated status was confirmed by viral culture prior to their use in the BSL-2 laboratory for establishment of the quality-control panel and for subsequent evaluation of commercial assays. Twelve other non-H7N9 influenza virus strains were also isolated, identified and cultured by the CNIC based on standard operating procedures, which included influenza A seasonal H1N1 (isolated before the 2009 pandemic), H1N1 pdm09, H3N2, H5N1 and influenza B viruses (S1 Table). After harvest, sufficient amounts of viral culture supernatants were immediately frozen at -70°C until use.

All of the viral culture supernatants were confirmed by RT-PCR and sequencing according to the protocol recommended by the WHO and the CNIC [13] (S1 File). Culture supernatants of the H7N9 virus were tested with the following PCR and sequencing primers (5’ to 3’): H7-F, GGCAACAGGAATGAAGAATGTTCC and H7-R, CACYGCATGTTTCCATTCTT for segment H7; N9-F, GTGATTCAGATAGACCCAGTAGCA and N9-R, ACTCCAGTCAGCGTTTAATACAAT for segment N9. The sequencing results were aligned with the influenza sequences deposited in the Global Initiative on Sharing Avian Influenza Data (GISAID) database (http://platform.gisaid.org) and in the Influenza Research database (IRD) (http://www.fludb.org/) to identify the correct types and subtypes of the viruses. The RT-PCR and sequencing analyses were independently performed using identical protocols in two different laboratories at the National Institutes for Food and Drug Control (NIFDC) and at the Beijing Institute of Radiation Medicine (BIRM).

#### Preparation of quality-control materials

The process of diluting and dispensing the viral cultures was conducted in a BSL-2 bio-safe protective environment, and freeze-drying was conducted in a GMP Grade D environment. Multipette stream electronic hand dispensers with Combitip bio-safe tips (Eppendorf, Germany) were used for the dispensing process. Approximately 10 mL of viral culture supernatants from the H7N9 virus strains A/Zhejiang/DTID-ZJU01/2013 and A/Zhejiang/DTID-ZJU02/2013 were designated P1 and P2, respectively, and were directly dispensed in 0.1-mL aliquots in screw-cap tubes. The culture supernatants of the remaining three H7N9 viruses (A/Shanghai/1/2013, A/Shanghai/2/2013 and A/Anhui/1/2013, designated P3, P4 and P5, respectively) were diluted 200-fold in lysis buffer RLT (Qiagen, Germany) and mixed for 30 min at room temperature to generate three bulk samples. Each bulk sample was then dispensed in 0.2-mL aliquots in screw-cap tubes. All of the liquid aliquots of the H7N9 virus (P1 to P5) were stored at -70°C. The culture supernatants of the non-H7N9 influenza virus strains were diluted 50- to 500-fold in lyophilization buffer to generate 12 bulk samples. Each bulk sample (0.5 mL) was placed in a 2-mL penicillin bottle and immediately processed for lyophilization. To avoid cross-contamination, the penicillin bottles filled with different types or subtypes of virus were freeze-dried according to routine procedures on separate days. The penicillin bottles were lightly fitted with rubber stoppers and were then lyophilized in a Genesis 25LE freeze-dryer (Virtis, USA). Finally, the penicillin bottles were crimp-sealed with aluminum rings and stored at -70°C. The procedures and parameters for a typical freeze-drying process are shown in S2 File.

#### Quantification of the quality-control panel

The complete DNA sequences of three viral segments, the HA segment of the H7N9 virus strain A/Zhejiang/DTID-ZJU01/2013 (GISAID accession number: EPI_ISL_139364), the MP segment of the influenza A H1N1 pdm09 virus strain A/California/07/2009 (GenBank accession number: FJ969537) and the NS segment of the influenza B virus strain B/Brisbane/60/2008 (GenBank accession number: CY115155), were commercially synthesized in pUC- or pBluescript-derived plasmid vectors by TAKARA Biotech. (Dalian, China). The sequences of the synthetic HA, MP and NS fragments were verified in our laboratory by PCR and sequencing using plasmid-specific primers. To reduce the plasmid background, PCR products with T7 promoter sequences were used as templates for subsequent in vitro transcription. Using the constructed plasmids as templates for PCR, the essential sequence of the T7 promoter were added upstream of the HA, MP and NS fragments using specific primers (S2 Table). Next, the PCR products were gel-extracted, photometrically quantified and transcribed into RNA using the MegaScript T7 in vitro transcription kit (Ambion, Life Technologies, USA). After RNase-free DNase I digestion, the RNA transcripts were purified using the Qiagen RNeasy kit (Qiagen, Germany) and quantified photometrically with a Nanodrop 2000 (Thermo, USA). The copy numbers of the in vitro RNA transcripts were calculated according to the following formula: RNA quantity (copies/μL) = [RNA concentration (ng/μL) * 10^−9^] * (6.02*10^23^) / [RNA length (nt) * 340]. Ten-fold serially diluted RNA transcripts were used to generate a standard curve for quantification of the quality-control panel based on the WHO-recommended real-time RT-PCR method [13].

#### Stability of the quality-control panel

Real-time stability evaluations were conducted 6 and 12 months after the panel was produced. All of the panel samples were extracted and tested in two independent experiments using in-house assays to monitor any increase in threshold cycle (Ct) values compared with the values at the time of production (0 months). An accelerated-degradation study was also conducted to evaluate the stability of the quality-control panel. The panel samples were placed at 4°C for 1 week and repeatedly freeze-thawed five times. After this treatment, the samples were refrozen at -70°C and then tested concurrently with the untreated samples stored at -70°C in two independent experiments using the CNIC real-time RT-PCR method. The differences in the Ct values between untreated and treated samples were then analyzed.

### RNA extraction and real-time RT-PCR

The commercial kits that were evaluated in this study were produced by Shanghai ZJ Bio-Tech Co., Ltd. (Liferiver), DAAN Gene Co., Ltd. of Sun Yat-sen University (DAAN) and Shenzhen Puruikang Biotech Co., Ltd. (Puruikang). They were randomly sampled from three successively produced lots of each assay by blinding the manufacturers and evaluators. Kit-related information was provided by the manufacturers and is summarized in Table 1. The RNA extraction reagents recommended by the manufacturers were used according to the instructions provided with the kits. Specifically, for the Liferiver and DAAN assays, the RNA extraction reagent was the Nucleic Acid Isolation Kit (Spin Column Method) produced by the kit manufacturer; for the Puruikang assay, it was the TIANamp Virus RNA kit (Spin Column Method) produced by Tiangen Biotech (Beijing) Co., Ltd. The RNA extraction processes for the three commercial assays were conducted manually. For the WHO-CNIC assay, RNA extraction was performed using the QIAamp Viral RNA kit (Qiagen, Germany) according to the manufacturer’s protocols. The real-time RT-PCR procedures and result interpretation for the commercial and WHO-CNIC assays were conducted strictly according to the kit instructions and WHO protocols [13] respectively, with two modifications: (1) during the analytical evaluation of the kits, suspicious results were considered to be positive without repeated testing (e.g., for the WHO-CNIC assay, a positive result was defined as a Ct value ≤ 38.0, and as a Ct value > 38.0 without repeated testing, while a negative result was defined as a reaction with no amplification curve); (2) the criteria for the internal control (human RNaseP) were not considered when interpreting the results of the analytical evaluation of the Puruikang assay, as the samples from chicken egg allantoic fluid did not contain detectable human material. The experimental procedures, result interpretation and quality-control criteria for each kit are documented in detail in S3 File.

**Table 1:** Characteristics of the three evaluated commercial diagnostic assays used to specifically detect avian influenza A (H7N9) viral RNA.

Assay	Liferiver	DAAN	Puruikang
Detection method	Taqman probe one-step real-time RT-PCR	Taqman probe one-step real-time RT-PCR	Complex probe one-step real-time RT-PCR
Targets detected per reaction^a^	2	1	1
Reactions needed per sample	1	2	2
Sample volume/reaction volume (l)	5/25	5/25	10/30
Compatible real-time PCR platform	ABI 7500^b^, Bio-Rad CFX96	ABI 7500^b^, ABI 7300, Roche Light Cycler 480	ABI 7500^b^, Bio-Rad CFX96, Roche Light Cycler 480
Fluorescence detection channels	FAM, VIC/HEX, Texred/Cal Red 610	FAM, VIC/HEX	FAM, VIC/HEX
Controls provided in the kit	Positive, negative and internal controls	Positive, negative and internal controls	Positive, negative and internal controls
Extraction of controls	All needed	Negative and internal control needed	All needed^a^
Positive control materials	Pseudovirus	Plasmid	Bacteriophage or pseudovirus
Type of internal control (IC) and controlled value	Competitive, exogenous Ct ≤ 43	Competitive, exogenous Ct ≤ 45	Noncompetitive, endogenousCt < 33
Target of IC	Influenza HA and NA gene^c^	Influenza HA and NA gene^c^	Human RnaseP gene
Internal control materials	Pseudovirus	Bacteriophage	NA
Time for PCR reaction	Within 2 hours	Within 2 hours	Within 2 hours
Number of PCR amplification cycles	45	45	40
Interpretation of the results	Ct ≤ 43: positive; No Ct: negative; 43 < Ct ≤ 45: suspect	Ct ≤ 42: positive; Ct > 42 or No Ct: negative	Ct ≤ 37: positive; No Ct: negative; 37 < Ct ≤ 40: suspect

a: Internal control is not included.

b: Real-time PCR platform used for evaluation.

c: The sequences targeted by internal-control probes are different from the ones targeted for the detection of avian influenza A (H7N9) viral RNA.

NA: Not applicable.

### Analytical evaluation using the quality-control panel

All of the samples in the quality-control panel were tested using the selected assays through the entire process of RNA extraction, amplification and signal detection, according to the instructions associated with each assay (S3 File). Before use, negative samples N1 to N12 were recovered using RNase-free PCR-grade (or equal) water. To determine the analytical sensitivity of the commercial kits and the WHO-CNIC assay, 10-fold dilutions of P1 were prepared to individually generate a series of working samples. RNA samples from specific dilutions pooled from several extractions were tested 20 times in each run to determine the limit of detection (LoD), which was defined as having at least 18 positive results among 20 replicates (90% probability). P2 was aliquoted at a 2500-fold dilution into 10 tubes for individual RNA extraction, amplification and signal detection, and the coefficient of variation (CV, %) of the 10 Ct values of the P2 samples was calculated for every product lot. In addition, P3, P4 and P5 had to be diluted by a factor of 10^4^ prior to use. The viral lysis buffers of the individual RNA extraction kits associated with each assay were used for the dilutions.

### Clinical evaluation using the patients’ specimens

Clinical evaluations of the three commercial assays were designed according to the individual situation (the assay characteristics, the accomplishment time of assay development, the available specimens and healthcare facility resources) and conducted separately; thus, the study site, the initial time and duration, the number of the specimens tested and other factors of each evaluation were not identical. Some essential requirements were set to be identical for the design of the clinical evaluations as follows. Positive samples were collected from patients with previously confirmed avian influenza A (H7N9) virus infections (H7N9-infected patients) [20], while negative samples were collected from patients with influenza-like illness (ILI) but who were not infected with avian influenza A (H7N9) virus (non-H7N9 ILI patients). The basic information on the patients participating in the study, including the age and gender, was documented. The positive samples from the participants for whom clear basic information was lacking were excluded from the analysis. The type of clinical specimen was throat swabs, sputum or tracheal aspirate. Positive samples sequentially collected from the same patient and the samples of different types simultaneously collected from the same patient were eligible for inclusion. It was recommended that the negative samples be further screened for other respiratory pathogens, such as influenza virus, respiratory syncytial virus, human coronavirus and *Mycoplasma*. The collection of the clinical specimens was conducted according to the standard operational protocol issued by CNIC. Once collected, the clinical specimens were transported on ice to the study sites and tested within 48 hours; if not tested within this period, they were stored at -70°C without frequent freeze-thawing before use. The clinical specimens were coded, and the laboratory technicians were blind to the positive/negative information about the specimens. The reference method used for all of the clinical evaluations was the WHO-CNIC assay, which is the key item of the official diagnostic standard for the confirmed H7N9-infected patients [20]. The experiments for the commercial and WHO-CNIC assays were performed strictly according to the kit instructions (S3 File) and WHO-CNIC protocols [13], respectively. The experimental procedures and the raw data from the test results were carefully documented. Repeated tests were performed for the samples that yielded discordant results between the commercial assay and the reference method. It was considered preferable to conduct DNA sequencing on the positive samples that had confirmed discordant results. Data management and statistical analysis (positive and negative agreements) were performed by independent personnel. To assure the quality of the clinical evaluations, personnel training and project monitoring were conducted by the investigators themselves.

#### (1) Evaluation of the DAAN assay

**Fig 1:**
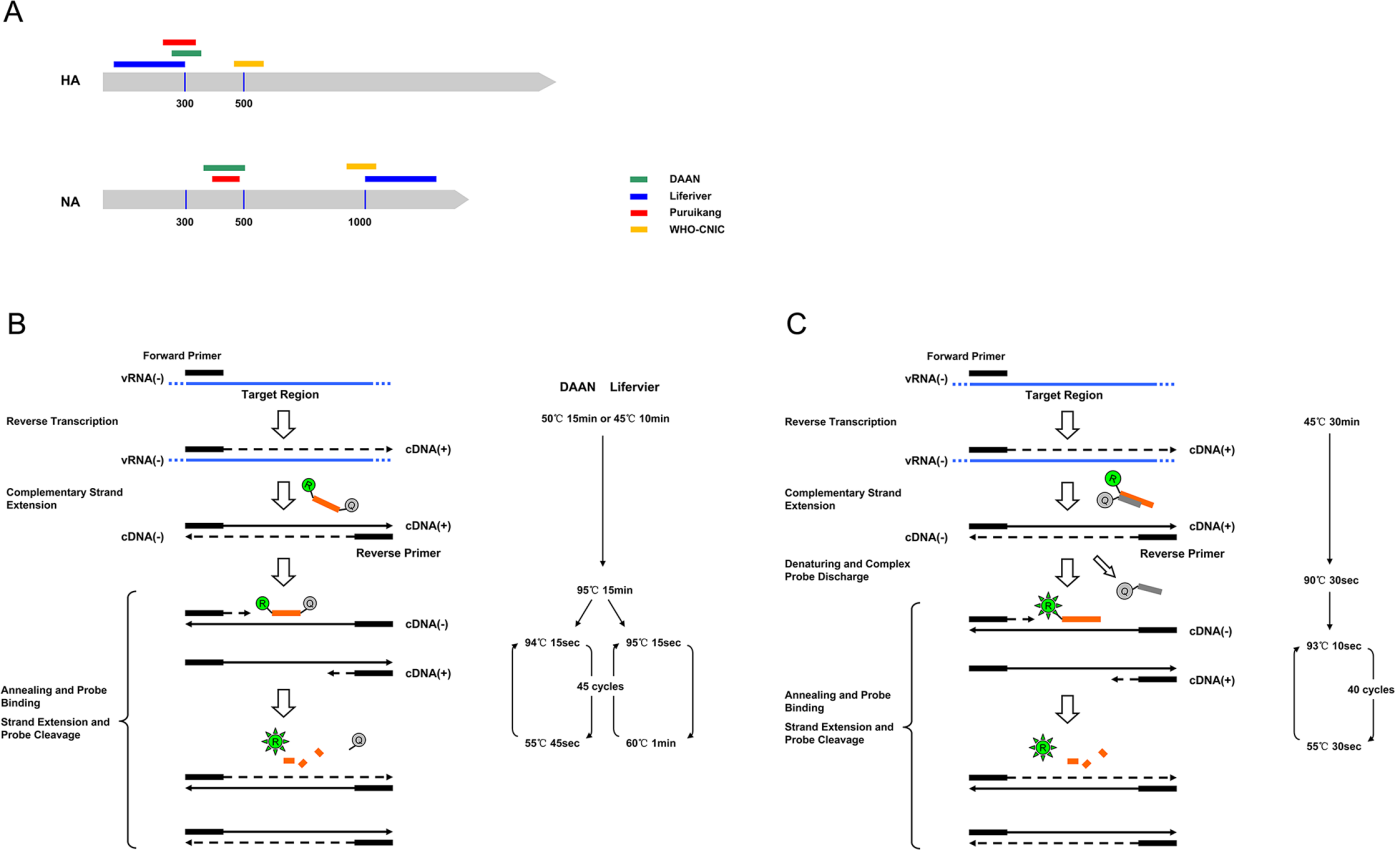
The geographic and temporal characteristics of the confirmed patients with human infection of avian influenza A (H7N9) virus from whom the positive clinical specimens were collected. The numbers of the enrolled patients from each location are presented individually for the DAAN assay (A), the Puruikang assay (B) and the Liferiver assay (C). The percentages of the clinical specimens that were collected during the first epidemic period (blue) and the second period (red) are illustrated by the pie charts in parts A and C of this figure. The line connecting the geographic map and the pie chart helps to identify the case number from the collection location related to the epidemic wave. The text and the following number above the line indicate the name of the collection location and the case number of each location. The dark-to-light red color on the map of mainland China indicates different levels of infection cases as of May 2014, which is interpreted in the lower left quarter of the figure.

A total of 1,286 H7N9 virus-positive and-negative samples were collected from 1,129 patients who were admitted to six hospitals or public health facilities (ZJU Hospital, Nanjing CDC, SPHCC, Zhejiang CDC, Guangdong CDC and Guangzhou CDC) between February 2013 and May 2014. The number and the percentage of the admitted patients are described based on the collection location and epidemic interval, respectively, in Fig 1A. Excluding 137 patients whose age and gender were unspecified (all were non-H7N9 ILI patients), the average age of the patients was 39.5 ± 25.4 years (ranging from 45 days to 95 years), and the male/female ratio was 1.37 (665/484). There were 409 positive samples collected from 252 H7N9-infected patients aged 58.7 ± 17.8 years (ranging from 2 to 88 years) and with a male/female ratio of 2.0 (258/131), including 249 throat swabs and 160 sputum specimens. The 877 H7N9 virus negative samples included 343 influenza virus-positive samples (subtypes H1N1pdm09, H3 and type B), 113 influenza virus-positive samples with an unknown subtype, 6 measles virus-positive samples, 10 *Mycobacterium tuberculosis*-positive samples, 10 *Mycoplasma*-positive samples and 5 *Chlamydia pneumoniae*-positive samples.

#### (2) Evaluation of the Puruikang assay

A total of 575 H7N9 virus-positive and-negative samples were collected from 545 patients who were admitted to four hospitals or public health facilities (Nanjing CDC, SPHCC, Zhejiang CDC and Jiangsu CDC) between February 2013 and May 2013 (within the first epidemic period). The patient cases and the collection locations are indicated in Fig 1B. Excluding 64 patients whose age and gender were unspecified (all were non-H7N9 ILI patients), the average age of these patients was 30.3 ± 30.1 years (ranging from 20 days to 101 years), and the male/female ratio was 1.65 (318/193). One hundred and twenty-two positive samples were collected using throat swabs from 92 H7N9-infected patients aged 61.5 ± 16.3 years (ranging from 7 to 89 years) with a male/female ratio of 3.1 (92/30), while 453 H7N9 virus negative samples included 82 influenza virus-positive samples (subtype H1N1pdm09, H3 and type B), 41 parainfluenza virus-positive samples, 35 respiratory syncytial virus-positive samples, 20 small RNA virus-positive samples, 19 adenovirus-positive samples, 8 bocavirus-positive samples and 6 human coronavirus-positive samples.

#### (3) Evaluation of the Liferiver assay

A total of 1,763 H7N9 virus-positive and-negative samples were collected from 1,652 patients who were admitted to three hospitals or public health facilities (ZJU Hospital, Hangzhou CDC, SPHCC and Guangdong CDC) between February 2013 and May 2014. The number and the percentage of the admitted patients are described based on the collection location and epidemic interval, respectively, in Fig 1C. Two hundred and ninety-four positive samples were collected from 183 patients with confirmed H7N9 virus infections, including 131 throat swabs and 96 sputum specimens or tracheal aspirates. The 1,469 H7N9 virus negative samples included 991 throat swabs and 478 sputum specimens.

### Statistical analysis

Analysis of variance (ANOVA) and t-test were performed using Prism 5 (GraphPad Software, USA), and statistical significance was defined as *p* < 0.05. Positive and negative agreements and the related 95% confidence interval (95% CI) were calculated using MedCalc 13.0 (MedCalc Software bvba, Belgium).

## Results

### Establishment of the quality-control panel

To analytically evaluate the commercial assays, a panel of quality-control materials consisting of five positive and twelve negative samples was established and underwent the processes of diluting, dispensing, freeze-drying, quantifying and evaluating stability. A set of samples (P1, P2, P3, P4 and P5) that were produced from a total of five different H7N9 virus strains were used to evaluate the analytical sensitivity, reproducibility and detection performance of the kits for diverse positive samples. Based on a modified WHO real-time RT-PCR method with a standard curve, the RNA copy numbers were determined to be 7.83 and 8.88 Log_10_ copies/μl for P1 and P2, respectively, while they were 6.93, 6.78 and 6.63 Log_10_ copies/μl for P3, P4 and P5, respectively. In addition, twelve negative samples (N1 to N12) that were positive for other influenza viruses were used to evaluate the analytical specificity of the assays; these samples included influenza A seasonal H1N1, H1N1 pdm09, H3N2, H5N1 and influenza B viruses (S1 Table). The virus strain names and RNA copy numbers for samples N1 to N12 are shown in S1 Table and were quantified using a modified WHO real-time RT-PCR technique with a standard curve targeting the MP gene (influenza A virus) or the NS gene (influenza B virus). Due to the relatively large amount of bulk solution (≥ 0.5 mL) required for the freeze-drying method and the limited amount of H7N9 viral culture obtained, samples P1 to P5 were prepared as liquid, while samples N1-N12 were lyophilized. At least 100 sets of the quality-control panel were produced to ensure consistency of evaluation and to meet long-term requirements for quality control of the assays. The stability study indicated that the samples constituting the panel were stable even when stored under degradation conditions of 4°C for 1 week, could be repeatedly freeze-thawed five times (S1 Fig), and were stable at -70°C for at least 12 months (S2 Fig). The packaging and component appearance of the panel are illustrated in S3 Fig.

### Characterization of the commercial kits

**Fig 2:**
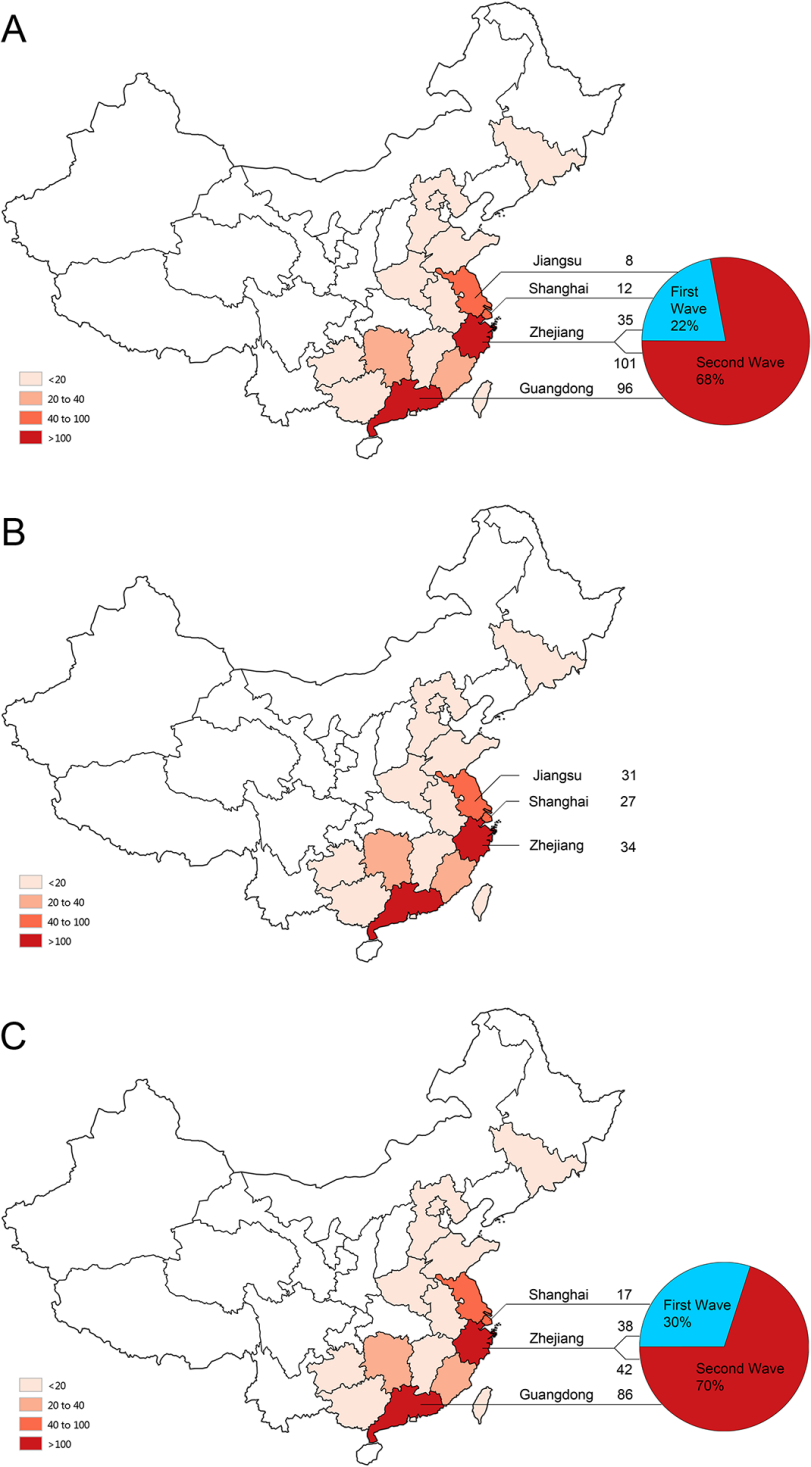
A schematic diagram of the target regions and basic principles of the commercial assays and WHO-CNIC method. (A) The relative position of the target regions of the commercial assays and WHO-CNIC method on the HA and NA genes of the avian influenza A (H7N9) virus. HA stands for the hemagglutinin gene, and NA stands for the neuraminidase gene. The green bar indicates the target region of the DAAN assay. The blue bar indicates the Liferiver assay target region. The red bar indicates the Puruikang assay target region. The yellow bar indicates the WHO-CNIC target region. The vertical line with the number below indicates the position of the viral genome, which is referred to the avian influenza A (H7N9) virus strain A/Zhejiang/DTID-ZJU01/2013(H7N9). The accession numbers are KJ633809 for HA and KJ633810 for NA. (B) The basic principles (left part) and key reaction parameters (right part) of the DAAN and Liferiver assays. vRNA(-), cDNA(+) and cDNA(-) indicate negative strand viral RNA segment, positive and negative strand complementary DNAs, respectively. The orange bar that ends with the green ‘R’ ball (reporter) and gray ‘Q’ ball (quencher) indicates the Taqman probe. The probe is supposed to be forward and bind the cDNA(-). (C) The basic principles (left part) and key reaction parameters (right part) of the Puruikang assays. The orange bar that ends with the green ‘R’ ball (reporter) indicates the fluorescent probe of the complex probe, whereas the gray bar with the gray ‘Q’ ball (quencher) indicates the quenching probe. The fluorescent probe is supposed to be forward and bind the cDNA(-).

The characteristics of the three diagnostic assays are listed in Table 1, and the appearance of the kits is shown in S4 Fig. In addition, the principles and basic reaction conditions for real-time RT-PCR specific to the commercial assays are illustrated in Fig 2. All of the assays were qualitative and were based on one-step real-time RT-PCR method; two assays (DAAN and Puruikang) were duplex and one assay (Liferiver) was triplex. Taqman hydrolysis probes were utilized in the real-time RT-PCR for the Liferiver and DAAN assays, while a previously developed complex-probe technique was used for the Puruikang assay [21]. Unlike the hydrolysis probe, which is a single short DNA strand labeled with the reporter and quencher at the 5’ and 3’ ends, respectively, the complex probe is composed of two short DNA strands labeled with the reporter and the quencher at the 5’ and 3’ ends individually. Ideally, if there is no target template in the samples, the two DNA strands hybridize with each other to form a tight complex during the annealing and extension thermal steps; thus, no fluorescence is emitted. The individual kits show different equipment compatibilities, as shown in Table 1; however, the ABI 7500 real-time PCR machine was supported by all of the assays and, hence, was used for all of the evaluations. In each of two assays (DAAN and Liferiver), the target sequences of the H7N9 virus and exogenously added internal controls were simultaneously amplified using one pair of primers, but the sequences of the probes targeting the internal controls differed from the ones used for the samples. The positive controls for the Liferiver and Puruikang assays were pseudovirus or bacteriophage, and they thus required extraction prior to conducting the real-time RT-PCR.

### Analytical performance evaluation of the commercial assays

The analytical sensitivity results for the commercial and WHO-CNIC assays are shown in Table 2. For both the H7 and the N9 targets, the LoD of all three commercial assays, based on the results of 20 replicates, was 2.83 Log_10_ copies/μl or 0.7 Log_10_TCID_50_/mL (10^5^-fold diluted P1 sample). The WHO-CNIC assay showed a better LoD for the H7 target (1.83 Log_10_ copies/μl) but the same N9 LoD as the commercial kits; however, the WHO-CNIC assay showed a higher frequency of positive replicates (15/20) when compared to the Liferiver (5/20) and Puruikang (8/20) assays. Interestingly, all of the commercial assays showed equal LoDs for the H7 and N9 targets of the H7N9 virus, while the LoDs for H7 and N9 in the WHO-CNIC assay were unequal. All of the P2 samples tested positive, and the CVs for the Ct values of the P2 samples were less than 5% for each lot of the three tested diagnostic assays, as indicated in Table 3. There were no obvious differences in the within- and between-lot reproducibility of different detection targets in each commercial assay. However, the between-lot CVs of the H7 and N9 Ct values in the WHO-CNIC assay were 3.4% and 5.2%, both of which were higher than the values obtained using the commercial kits (ranging from 0.7% to 2.8% for H7 and from 1.1% to 4.5% for N9). Samples P3, P4 and P5 (10^4^-fold diluted) were correctly detected as positive, indicating that the commercial assays had the ability to identify diverse H7N9 viruses. The results for the diluted P3, P4 and P5 samples showed no statistically significant differences between the Ct values for the H7 and N9 targets (Table 4). In addition, the N1 to N12 samples were correctly detected as negative by the commercial kits, indicating that there was no cross-reactivity with the H1N1 pdm09, seasonal H1N1, H3N2, H5N1 or influenza B viruses. All of the tests of the lysis buffers used for dilution produced negative results.

**Table 2:** The analytical sensitivity of commercial products for the specific detection of avian influenza A (H7N9) viral RNA.

		Positive results^a^ /Total tests (average Ct values^b^)							
		Liferiver		DAAN		Puruikang		WHO-CNIC	
P1 dilution	Equivalent RNA log_10_ copies/μl	H7	N9	H7	N9	H7	N9	H7	N9
1:10 ^1^	6.83	3/3 (25.0)	3/3 (26.2)	3/3 (19.0)	3/3 (21.2)	3/3 (20.9)	3/3 (20.5)	3/3 (17.4)	3/3 (18.3)
1:10 ^2^	5.83	3/3 (25.9)	3/3 (27.6)	3/3 (22.2)	3/3 (24.4)	3/3 (23.5)	3/3 (23.4)	3/3 (20.8)	3/3 (21.8)
1:10 ^3^	4.83	3/3 (27.7)	3/3 (28.0)	3/3 (26.7)	3/3 (27.7)	3/3 (27.1)	3/3 (27.1)	3/3 (24.4)	3/3 (25.3)
1:10 ^4^	3.83	3/3 (29.4)	3/3 (30.6)	20/20 (30.3)	20/20 (30.7)	3/3 (30.3)	3/3 (30.3)	3/3 (28.2)	3/3 (28.9)
1:10 ^5^	2.83	19/20 (32.5)	19/20 (33.9)	20/20 (34.0)	20/20 (35.4)	20/20 (33.0)	19/20 (34.2)	20/20 (31.7)	20/20 (31.8)
1:10 ^6^	1.83	5/20 (35.3)	5/20 (35.9)	9/20 (36.1)	15/20 (39.8)	13/20 (35.7)	8/20 (37.2)	18/20 (35.2)	15/20 (37.7)
1:10 ^7^	0.83	1/20 (35.9)	0/20 (0)	5/20 (40.7)	0/20 (0)	1/20 (38.4)	1/20 (38.4)	4/20 (36.4)	1/20 (37.7)
Lysis Buffer	0.00	0/3 (0)	0/3 (0)	0/3 (0)	0/3 (0)	0/3 (0)	0/3 (0)	0/3 (0)	0/3 (0)

a: Susceptible positive results were considered to be positive without repeated testing.

b: Average Ct values of the test results having a signal curve with a correct shape.

**Table 3:** The within- and between-lot reproducibility of the commercial and WHO-CNIC assays for the specific detection of avian influenza A (H7N9) viral RNA.

	Coefficient of Variation (CV, %)/Average of the Ct values							
Assay	Liferiver		DAAN		Puruikang		WHO-CNIC^a^	
Target	H7	N9	H7	N9	H7	N9	H7	N9
Lot 1	1.3/28.0	2.6/29.3	4.0/26.0	1.1/28.1	0.8/25.9	1.4/28.0	3.2/25.1	3.5/25.5
Lot 2	2.1/28.5	2.3/29.9	1.4/26.8	0.9/28.5	1.2/25.9	1.1/26.1	4.5/24.4	2.2/23.9
Lot 3	2.2/28.0	2.8/29.4	1.2/27.5	1.6/29.1	1.6/25.6	1.6/25.8	1.8/26.1	2.1/26.5
Between-Lot	1.0/28.2	1.1/29.5	2.8/26.8	1.8/28.6	0.7/25.8	4.5/26.6	3.4/25.2	5.2/25.3

a: For the WHO-CNIC assay, one lot of the assay was treated as an independent test using a different lot of RT-PCR reagent.

**Table 4:** Results of the commercial assays for the detection of diverse avian influenza A (H7N9) viral culture samples (P3, P4 and P5).

		Average of the Ct values ± Standard deviation^a^					
		Liferiver		DAAN		Puruikang	
Sample	Equivalent RNA log_10_ copies/l	H7	N9	H7	N9	H7	N9
P3	2.93	32.5 ± 0.6	31.8 ± 0.6	31.2 ± 1.2	32.1 ± 0.2	32.0 ± 1.0	31.7 ± 0.7
P4	2.78	33.4 ± 0.6	32.7 ± 0.6	30.7 ± 0.2	33.7 ± 1.2	32.6 ± 0.6	30.9 ± 3.4
P5	2.63	32.6 ± 1.6	31.9 ± 0.6	31.1 ± 5.9	31.0 ± 2.0	31.9 ± 0.8	32.5 ± 0.5

a: Calculated from the results of triplicate samples within a single run.

### Clinical evaluation of the commercial assays

The results of the three clinical trials are shown in Table 5. Among 294 clinical samples that were determined to be positive using the WHO-CNIC assay, 291 were also found to be positive using the Liferiver assay, and all the known negative samples were correctly identified as negative. Thus, the positive and negative agreement values were 99% and 100%, respectively. For the DAAN assay, the positive and negative agreement values were 98.5% (403/409) and 99.9% (876/877), respectively, with respect to the results of the WHO-CNIC assay. The six positive samples with results that were discordant between the DAAN assay and the reference assay were all throat-swab samples; they were subjected to RT-PCR and sequencing for confirmation. Among these discordant samples, three showed negative results in the RT-PCR and sequencing analysis, indicating that there was an extremely small amount of target RNA in these samples. Both the positive and negative agreements were 100% for the Puruikang assay.

**Table 5:** Results of the clinical evaluation of commercial assays using the WHO-CNIC assay as a reference method.

	No. of samples with positive results			No. of samples with negative results				
	Commercial assay	WHO-CNIC assay	Positive agreement (95% CI)	Commercial assay	WHO-CNIC assay	Negative agreement (95% CI)	Total No. of samples tested	No. of H7N9-infected patients
DAAN	403	409	98.5% (89.2%, 100.0%)	876	877	99.9% (93.4%, 100.0%)	1286	252
Puruikang	122	122	100.0% (83.0%, 100.0%)	453	453	100.0% (91.0%, 100.0%)	575	92
Liferiver	291	294	99.0% (87.9%, 100.0%)	1469	1469	100.0% (95.0%, 100.0%)	1763	183

CI: confidence interval.

## Discussion

Influenza A virus, a single, negative-strand RNA virus that belongs to the *Orthomyxoviridae*, commonly causes mild flu symptoms but can also cause pneumonia and even, in some cases, acute respiratory distress syndrome (ARDS) or multi-organ failure. Due to frequent sequence alterations and segment exchanges, this virus has been responsible for several pandemics or outbreaks in the past century, including H1N1 in 1918, H2N2 in 1957, the H3N2 pandemic in 1968, the H1N1 pandemic in 1977, and the recent H1N1 pandemic in 2009 and H5N1 outbreak in 2013 in China. In February 2013, human infections with a novel H7N9 virus were first reported in China [2]. Unlike the previous epidemic avian H5N1 influenza A virus, animal infection with this virus can be overlooked due to its low pathogenicity in poultry [5] during the early stage of an outbreak. Thus, the prevention and control of the resulting disease are difficult. More importantly, there has been evidence that, due to its re-emergence in China at the end of 2013 [16], the virus has continued to evolve to have higher adaptivity and pathogenicity in humans, increasing the possibility of a pandemic [22]. Thus, rapid and sensitive tools for the detection of this virus are urgently needed. Viral isolation and culture methods lack sensitivity and are time-consuming, thus leading to diagnostic delays. Rapid tests for viral antigens are also of low sensitivity and are difficult to develop quickly due to the requirement for virus-specific antibody production. In addition, detection of specific antibodies in infected patients using techniques such as enzyme-linked immunosorbent assay (ELISA) is not suitable for early diagnosis of the disease, as the antibodies require weeks to emerge. However, molecular assays based on real-time PCR are suitable diagnostic and surveillance tools, and their development is urgently needed to control the current outbreaks and to prevent the spread of this deleterious virus. To respond in an urgent manner to its emergence and to prepare for a potential pandemic of this novel influenza virus in the future, the CFDA approved three commercial real-time RT-PCR assays for specifically detecting avian influenza A (H7N9) viral RNA in May and July, 2013 for emergency use [17]. Additionally, in February 2014, the US FDA issued emergency use authorization for a molecular assay to specifically detect avian influenza A (H7N9) virus [23].

Before diagnostic assays are used in healthcare facilities, the effectiveness of the assays must be well evaluated. Plasmid DNA and cDNA are stable materials that are widely used for analytical evaluation but are not suitable for assays used to detect RNA samples, as the reverse transcription step cannot be characterized. In vitro-transcribed RNA is a commonly used material to evaluate the performance of RNA detection assays; however, it cannot be used to validate the RNA extraction process of the assays. To simulate the detection process of clinical specimens, all of the components of the quality-control panel established in the present study were produced from inactivated culture supernatants of viral strains so that the RNA extraction step could be tested. Furthermore, the biological and molecular features of the panel samples, such as the virus titer, copy number and gene sequences, were well characterized. The panel was also validated to be sufficiently stable for long-term quality-control requirements.

Using the newly established quality-control panel, three commercial diagnostic products were evaluated for their analytical performance in terms of sensitivity, specificity, reproducibility and inclusiveness of diverse positive samples. Sequence diversity among H7N9 viruses isolated from different patients has been observed in previous studies [3, 22], and thus the ability to identify different H7N9 viruses is an important factor in the quality of diagnostic assays. Five different H7N9 viruses in the panel and additional positive clinical specimens collected from more than 90 infected patients could be successfully detected, thus validating the sensitivity and inclusiveness of the assays. In addition, other common influenza A (H1N1 pdm09, seasonal H1N1 and H3N2) and influenza B viruses in humans were tested to evaluate the specificity of the products. For each seasonal H1N1, H3N2 and influenza B virus, several strains were included, which were isolated from different locations and epidemic periods and thus represented diverse circulating viruses. Due to the similarity of their severe symptoms, two strains of another avian influenza virus, H5N1, were also included as negative panel samples. Excellent negative agreement with the reference WHO-CNIC assay was achieved for the negative panel samples (100% for all commercial assays) and for clinically negative samples from ILI patients (>99% for all commercial assays). In comparison to the WHO-CNIC assay, lower values were obtained for the CV within and between lots for all of the assays, which suggested that the evaluated diagnostic assays reproducibly and reliably detected avian influenza A (H7N9) virus.

The avian influenza A (H7N9) virus is considered to replicate preferentially in the lower respiratory tract mucosal cells due to the presence of more numerous 2,3-linked sialic acid receptors [6]; consequently, upper respiratory tract specimens may not contain a sufficient viral load to be detectable, even during peaks of infection with rapid clinical deterioration [24]. The limited number of viral particles in the collected clinical samples requires suitably sensitive molecular detection assays in clinical settings. Unfortunately, a recent study reported that all of the evaluated commercial assays that are currently used in frontline laboratories had LoD values that were higher than 3.0 Log_10_TCID_50_/mL, indicating that they are consistently less sensitive than in-house real-time RT-PCR assays [25]. Furthermore, the commercial multiplex xTagRVP assay (Luminex Molecular Diagnostics, USA) was approximately 200-fold less sensitive than the WHO-CNIC method. This difference may be due to reliance of commercial assays on the detection of the conserved region of the M segment of the influenza virus without any optimization for the detection of the novel H7N9 virus, which harbors nucleic acid variations within the conserved region. However, the results of our study showed that the commercial assay LoD for the detection of the novel avian H7N9 virus was 2.83 Log_10_ copies/μl (0.7 Log_10_TCID_50_/mL, A/Zhejiang/DTID-ZJU01/2013). This LoD is generally comparable to the reference method (WHO-CNIC assay), although the commercial assays were slightly (10-fold) less sensitive than the reference method for the detection of the H7 target. The results obtained in the clinical trials confirmed the small difference in sensitivity between the commercial and WHO-CNIC assays, as they showed greater than 97% positive agreement for all of the commercial assays. Notably, the Liferiver assay, which simultaneously detects three targets (H7, N9 and internal control), added convenience to the detection method (especially for sample addition) while showing LoD values that were identical to the other two commercial assays, thus giving it an advantage.

As observed in some recently published studies [14, 26, 27], the LoD of the H7 target in newly developed molecular assays for specifically detecting the H7N9 virus is likely to be better than that of the N9 target. A similar result was documented in a previous study of the avian influenza H5N1 virus, demonstrating that assays detecting the H5 segment were more sensitive than those detecting N1 [28]. Kalthoff et al. evaluated two assays for analytical sensitivity using serially diluted RNA from the H7N9 virus A/Anhui/1/2013 and reported that both their own assay and the assay developed by Corman et al. [29] could detect samples at a dilution of 10^8^ for the H7 target, while neither assay could detect N9 [26]. The results of other studies have indicated that the WHO-CNIC assay was 10- to 100-fold more sensitive for the detection of the H7 segment than for the N9 segment [11, 14]. Consistent with the research described above, the results of our study showed that the WHO-CNIC assay was 10-fold more sensitive for the detection of the H7 segment than for N9. However, all of the commercial assays exhibit identical analytical sensitivities for the H7 and N9 targets based on detection of 10-fold serial dilutions of the H7N9 virus (sample P1), which is confirmed by the findings that the Ct values of the detection results of samples P3 to P5 were not significantly different between the H7 and N9 targets. The balanced LoDs for the H7 and N9 segments in the commercial kits are more likely to facilitate the interpretation of results in regular clinical practice. The explanation for the different LoDs observed for the HA and NA segments is complicated. It is possible that the NA segment naturally has fewer copies of transcripts than the HA segment, that it is easily degraded in clinical specimens or that it contains more complex sequences that may result in difficulty in primer/probe design compared to the HA segment; however, these hypotheses require further investigation for clarification.

Following the outbreak of human infections caused by avian influenza A (H7N9) virus, many molecular assays have been developed by laboratories and have been evaluated using clinical specimens. However, the H7N9 virus-positive clinical samples used in most studies typically number fewer than 20 and were collected from a single location during the first wave of the disease [10–12, 14, 15, 27] because these studies were conducted during the early stages of the H7N9 outbreak when H7N9-infected patients were scarce. Notably, the influenza virus is an RNA virus that tends to exhibit sequence changes and segment reassortment. Indeed, more genetic diversity was found among H7N9 virus strains during the initial outbreak of the disease; one of the earliest identified strains, A/Shanghai/1/2013, showed particularly high diversity in comparison to other H7N9 viruses [2, 3, 30]. After the first wave of the epidemic occurred in China in February 2013, human infection with the avian influenza A (H7N9) virus re-emerged in October 2013 as a second wave of the epidemic [16, 31]. Sequence differences were also observed between viruses collected during each wave [16, 22, 31]. In accordance with the re-emergence and continuous transmission of the disease, additional distinct lineages or genotypes were identified across provinces and epidemic periods, thus increasing the genetic heterogeneity of the virus [22, 30, 32]. Furthermore, it has been reported that, due to dynamic reassortments with local avian influenza H9N2 viruses, H7N9 viruses with different lineages are distributed geographically across provinces. For instance, there are Guangdong/Hong Kong-derived strains that have been observed only in Guangdong province [22]. Because of the observed sequence diversity of the H7N9 virus, a suitable assay evaluation requires clinical specimens with good geographical and temporal representation. In our study, more than 100 clinical samples from confirmed H7N9 virus-infected patients were tested using each assay. The clinical specimens used for evaluating the commercial kits were collected from three (Puruikang and Liferiver) or four (DAAN) major epidemic regions of China (Zhejiang, Guangdong, Jiangsu province and Shanghai city, each with more than 40 cumulative cases) and at intervals encompassing two epidemic waves of the disease (DAAN and Liferiver). Particularly for the DAAN assay, the clinical specimens were collected from 252 infected patients, thus including more than half of the total confirmed cases reported to date in China. The successful evaluation of the patient samples further supported the use of these assays in clinical laboratories.

Viral loads of the H7N9 virus have been recognized as significantly lower in throat swab samples from the upper respiratory tract than in sputum and tracheal aspirates from the lower respiratory tract, hampering the usefulness of the rapid antigen detection assay for diagnosing H7N9 virus [24, 33, 34]. Thus, clinical samples with different specimen types, especially throat swab, must be used to validate the effectiveness of the assays. More than 100 throat swabs were assessed in the present study to evaluate the sensitivity of the commercial assays for the detection of H7N9 virus in upper respiratory tract specimens (DAAN, 249; Puruikang, 122; Liferiver, 131). More than 98% of the positive samples defined by the reference method were correctly detected by all of the commercial assays, and only six and three samples from H7N9-infected patients yielded discordant results between the reference method and the DAAN and Liferiver assays, respectively. Consistent with previous findings, the positive samples that were not detected by the DAAN assay were all throat swab samples, indicating a low copy level of H7N9 virus in the upper respiratory tract. The observed high sensitivities of the commercial assays in our study suggested only a limited detrimental effect of throat swab specimens on their detection. However, because the previously reported average Ct value for a throat swab sample was approximately 35 [34], which is close to the cutoff of each assay, caution should be used when working with this type of specimen.

It has been previously reported that inhibitory substances are present in clinical samples [35, 36]; such substances may still be active after the process of nucleic acid extraction and interrupt enzyme-based PCR amplification and can reduce the sensitivity of the PCR reaction, leading to false-negative results. An internal control, which is added to each sample prior to the nucleic acid extraction, serves to monitor inhibitory factors in the extracted specimen and to ensure sufficient PCR amplification. Consequently, such internal controls are required in PCR-based assays to decrease the occurrence of false-negative results and to increase the sensitivity of the assay for the detection of the target nucleic acid in clinical samples, especially in the presence of a complicated matrix, such as plasma and sputum. In the present study, we found that, in contrast to the WHO-CNIC assay and to certain published assays that detect the H7 and N9 targets in separate reactions (one target per tube), the evaluated commercial duplex or triplex assays detected the internal controls as well as H7 and/or N9 in one tube. The simultaneous detection of the internal-control targets and viral RNA in one reaction can lead to better control of false-negative results; however, if designed improperly, internal controls may also limit the ability to detect low amounts of viral RNA, especially in comparison to simplex assays. Thus, the simultaneous detection of the internal control in the commercial assays may be one explanation for the present observation that, for the H7 target, the analytical sensitivities of all of the commercial assays were worse than that of the WHO-CNIC assay. However, given that the commercial assays will be distributed broadly and used in routine clinical practice without special support, the advantages of the internal control for reliable and stable quality control exceed its disadvantages in terms of the potential loss of sensitivity. In fact, an internal control using the MS2 bacteriophage was incorporated into the molecular assay used to specifically detect the avian influenza A (H7N9) virus that was authorized by the US FDA [37], demonstrating the essential role of the internal control for the use of diagnostic assays in the clinical laboratory. Additionally, the external positive and negative controls contained in the kits, which underwent the whole process of detection (including RNA extraction), monitored the false negative results caused by reagent failure (e.g., primer/probe degradation or enzyme inactivation) and operation errors, as well as false-positive results caused by reagents and/or environmental contaminants. These external controls work together with the internal control described above, resulting in stringent and sustained quality control for commercial assays.

The three evaluated commercial assays exhibit similarities regarding the intended use and their analytical and clinical performance. However, certain differences are observed, including the type of real time fluorescent PCR technique, the target region of the primers and probes, and the reverse transcription (RT) and PCR settings. First, the basic principles of the three assays are the same, namely, one-step real time RT-PCR; however, two different types of probe techniques were utilized. Taqman hydrolysis probes were incorporated in the Liferiver and DAAN assays, whereas a complex probe was used in the Puruikang assay. As illustrated in Fig 2, when the complex probe hybridizes, the quencher is closer to the reporter; thus, the latter’s fluorescence is more effectively inhibited than that of the Taqman probe. Thus, the complex probe generally exhibits reduced background fluorescence compared with the traditional Taqman hydrolysis probe, leading to increased analytical sensitivity. This finding may explain why the Puruikang assay uses fewer amplification cycles than two other assays (40 cycles vs. 45 cycles) but exhibits the same LOD. Second, the reverse transcription (RT) and PCR settings may also influence the analytical and clinical performance of the assays; however, different combinations of reaction parameters ultimately yield similar performances for the three commercial assays. For example, the increased duration of the RT reaction of the Puruikang assay compared with the Liferiver and DAAN assays (30 minutes vs. 10 minutes/15 minutes) likely increases its LOD, but this potential increase was eliminated by the subsequently reduced extension time (30 seconds vs. 45 seconds/ 60 seconds) and cycle numbers (40 cycles vs. 45 cycles). Third, the detection regions of the three commercial assays and the WHO-CNIC method were located on both the HA and NA genes of avian influenza A (H7N9) virus but were found to be more or less different from each other. Although the positions of the target regions in each gene were diverse, a certain consensus was noted among the commercial assays, which may account for the equal LODs observed among the assays. All three commercial assays targeted the region around the 300-nt position of the HA gene, and two commercial assays (DAAN and Puruikang) targeted the region around 400 nt of the NA gene. For the WHO-CNIC method, the target region was beyond the consensus position of the commercial assay. The difference in the target regions between WHO-CNIC and the commercial assays may explain the enhanced sensitivity of the WHO-CNIC method for the detection of the viral H7 segment.

Our study had two limitations. In a recent study, it was found that several molecular assays that were recently developed to detect avian influenza A (H7N9) virus also display cross-reactivity with avian or other animal H7- or N9-subtype influenza viruses other than the current human-infecting H7N9 virus [26]. Other H7- or N9-subtype influenza viruses were not included in our quality-control panel or clinical specimens. Thus, our study cannot exclude the possibility that the commercial assays have cross-reactivity with these H7- or N9-subtype animal influenza viruses, which should be investigated in a future study. However, as the results of the commercial assays will be interpreted as avian influenza A (H7N9) virus RNA-positive only when both H7 and N9 are positive, and as the intended use of the assays is not to test the animal samples with H7- or N9-subtype influenza viruses, the undetermined cross-reactivity may not hamper the application of the assays for current patient samples. Another limitation of our study is that stabilized human materials were not present in the samples of the quality-control panel, thus preventing us from evaluating the efficacy of the endogenous internal control in the Puruikang assay.

In conclusion, this study first established a panel of quality-control materials that was then used to conduct an analytical evaluation of commercial kits. In addition, the clinical performance of each kit was validated using a large number of patient specimens with diverse characteristics and good representativeness. Therefore, our study provides comprehensive evidence regarding the performance of commercial diagnostic assays for the specific detection of the avian influenza A (H7N9) virus. The high performance of these commercial diagnostic assays warrants their application in clinical settings. We believed that this study will benefit preparedness for the re-emergence or even potential pandemic of this virus by demonstrating the detection efficacy of the commercial assays and may facilitate quality improvement of such assays. Although it was established on an urgent basis, the quality-control panel used in the present study is indispensable for the quality evaluations of similar products and can be acquired upon request from the National Institutes of Food and Drug Control, China.

## Supporting Information

S1 Fig Stability of the quality-control panel against accelerated degradation. Open squares indicate results obtained for samples stored at -70°C, which were treated as controls. Filled circles indicate the results for samples stored at 4°C for 1 week. Red triangles indicate the results for samples that were repeatedly freeze-thawed five times.
(JPG)

S2 Fig Real-time stability of the quality-control panel. Yellow columns indicate the results for samples at the time of production, while red and blue columns indicate the results obtained for samples at 6 and 12 months after production, respectively. The Ct values of two independent tests are illustrated as the mean (column top) and standard deviation (bar).
(JPG)

S3 Fig Appearance of the quality-control panel and its components. Blue-capped penicillin bottles (labeled N1-N12) contain lyophilized viral cultures of non-H7N9 influenza viruses, while vials with orange or purple caps (labeled P1-P5) contain liquid viral cultures of avian influenza A (H7N9) virus. After lyophilization, the material in the bottles appeared to be milky-white, loose, thick pie-shaped.
(JPG)

S4 Fig Appearance of the commercial assays. Front row from left to right: the detection kits of Liferiver, DAAN, and Puruikang. Back row from left to right: the RNA extraction reagents coupled with the detection kits of Liferiver, DAAN, and Puruikang.
(JPG)

S1 File CNIC protocol for determining influenza virus type or subtype in samples. (DOC)

S2 File Procedures and parameters used to freeze-dry the quality-control panel. (DOC)

S3 File Instructions for the diagnostic assays used to specifically detect the avian influenza A (H7N9) viral RNA. (PDF)

S1 Table Influenza virus strains and their final concentrations in the reference panel. (DOC)

S2 Table Primers used for the addition of the T7 sequence by PCR amplification. (DOC)
