# Correction: Evaluation of Commercial Diagnostic Assays for the Specific Detection of Avian Influenza A (H7N9) Virus RNA Using a Quality-Control Panel and Clinical Specimens in China

**DOI:** 10.1371/journal.pone.0140089

**Published:** 2015-10-02

**Authors:** Dawei Shi, Shu Shen, Xingliang Fan, Suhong Chen, Dayan Wang, Changgui Li, Xing Wu, Lili Li, Dongting Bai, Chuntao Zhang, Junzhi Wang

The images for Figs 1 and 2 are incorrectly switched. The image that appears as Fig 1 should be Fig 2, and the image that appears as Fig 2 should be Fig 1. The figure captions appear in the correct order. Please view the correct Fig 1 and caption and correct Fig 2 and caption here.

**Fig 1:**
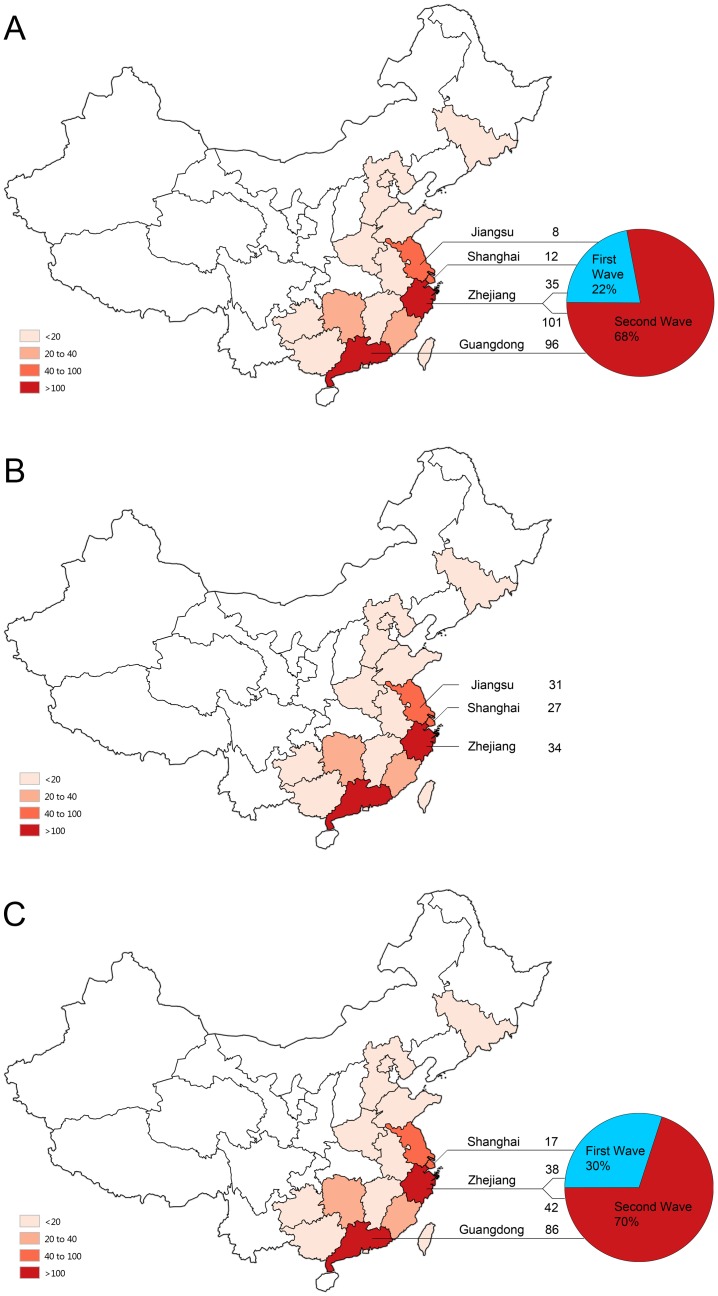
The geographic and temporal characteristics of the confirmed patients with human infection of avian influenza A (H7N9) virus from whom the positive clinical specimens were collected. The numbers of the enrolled patients from each location are presented individually for the DAAN assay (A), the Puruikang assay (B) and the Liferiver assay (C). The percentages of the clinical specimens that were collected during the first epidemic period (blue) and the second period (red) are illustrated by the pie charts in parts A and C of this figure. The line connecting the geographic map and the pie chart helps to identify the case number from the collection location related to the epidemic wave. The text and the following number above the line indicate the name of the collection location and the case number of each location. The dark-to-light red color on the map of mainland China indicates different levels of infection cases as of May 2014, which is interpreted in the lower left quarter of the figure.

**Fig 2:**
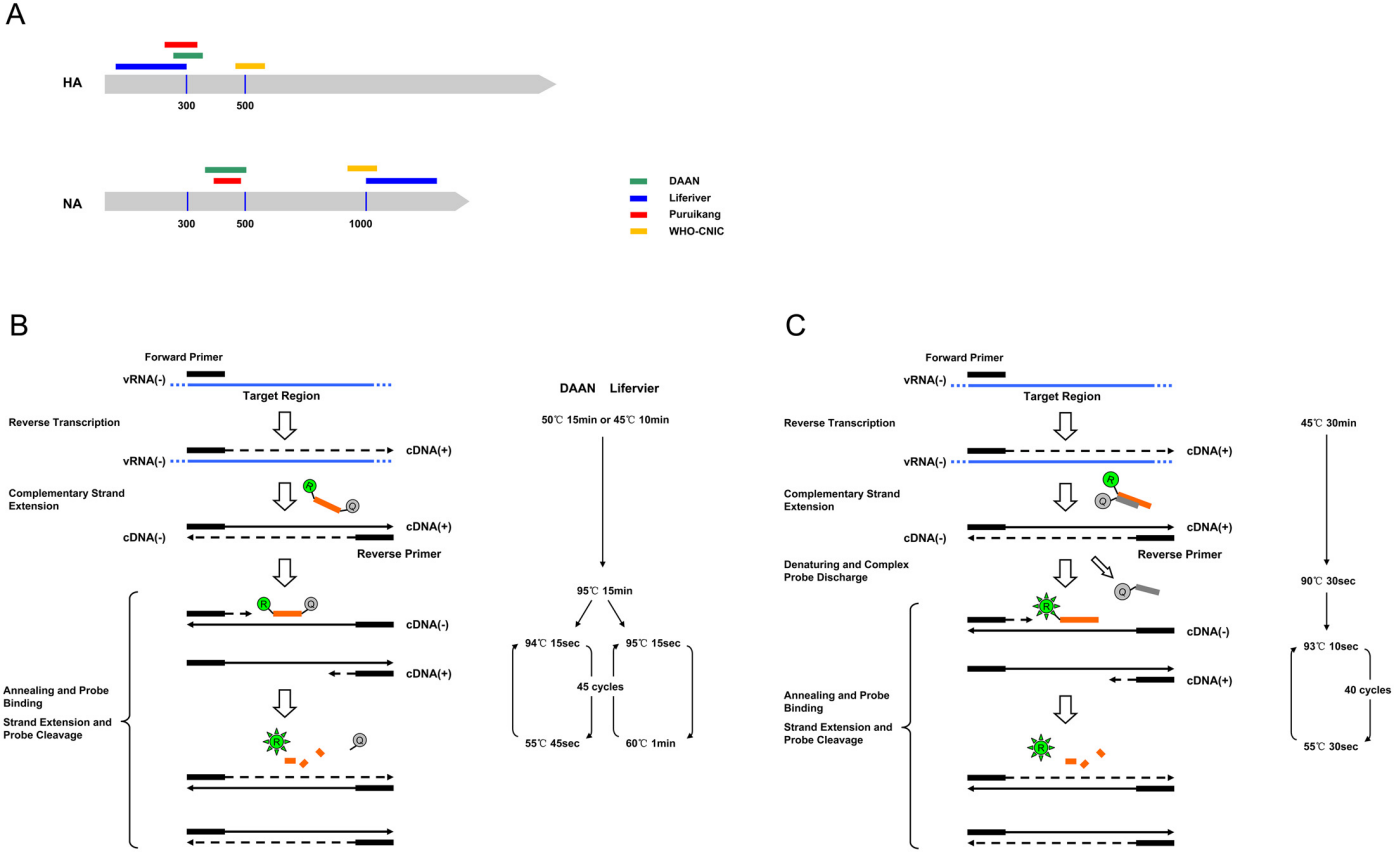
A schematic diagram of the target regions and basic principles of the commercial assays and WHO-CNIC method. (A) The relative position of the target regions of the commercial assays and WHO-CNIC method on the HA and NA genes of the avian influenza A (H7N9) virus. HA stands for the hemagglutinin gene, and NA stands for the neuraminidase gene. The green bar indicates the target region of the DAAN assay. The blue bar indicates the Liferiver assay target region. The red bar indicates the Puruikang assay target region. The yellow bar indicates the WHO-CNIC target region. The vertical line with the number below indicates the position of the viral genome, which is referred to the avian influenza A (H7N9) virus strain A/Zhejiang/DTID-ZJU01/2013(H7N9). The accession numbers are KJ633809 for HA and KJ633810 for NA. (B) The basic principles (left part) and key reaction parameters (right part) of the DAAN and Liferiver assays. vRNA(-), cDNA(+) and cDNA(-) indicate negative strand viral RNA segment, positive and negative strand complementary DNAs, respectively. The orange bar that ends with the green ‘R’ ball (reporter) and gray ‘Q’ ball (quencher) indicates the Taqman probe. The probe is supposed to be forward and bind the cDNA(-). (C) The basic principles (left part) and key reaction parameters (right part) of the Puruikang assays. The orange bar that ends with the green ‘R’ ball (reporter) indicates the fluorescent probe of the complex probe, whereas the gray bar with the gray ‘Q’ ball (quencher) indicates the quenching probe. The fluorescent probe is supposed to be forward and bind the cDNA(-).

## References

[1] Shi D, Shen S, Fan X, Chen S, Wang D, Li C, et al (2015) Evaluation of Commercial Diagnostic Assays for the Specific Detection of Avian Influenza A (H7N9) Virus RNA Using a Quality-Control Panel and Clinical Specimens in China. PLoS ONE 10(9): e0137862 doi: 10.1371/journal.pone.0137862 26361351 10.1371/journal.pone.0137862 PMC4567293

