# Supplementary figures and images for: Evaluation of Commercial Diagnostic Assays for the Specific Detection of Avian Influenza A (H7N9) Virus RNA Using a Quality-Control Panel and Clinical Specimens in China
Source: PLoS One. 2015 Sep 11;10(9):e0137862. doi: 10.1371/journal.pone.0137862 (PMC4567293; doi:10.1371/journal.pone.0137862)

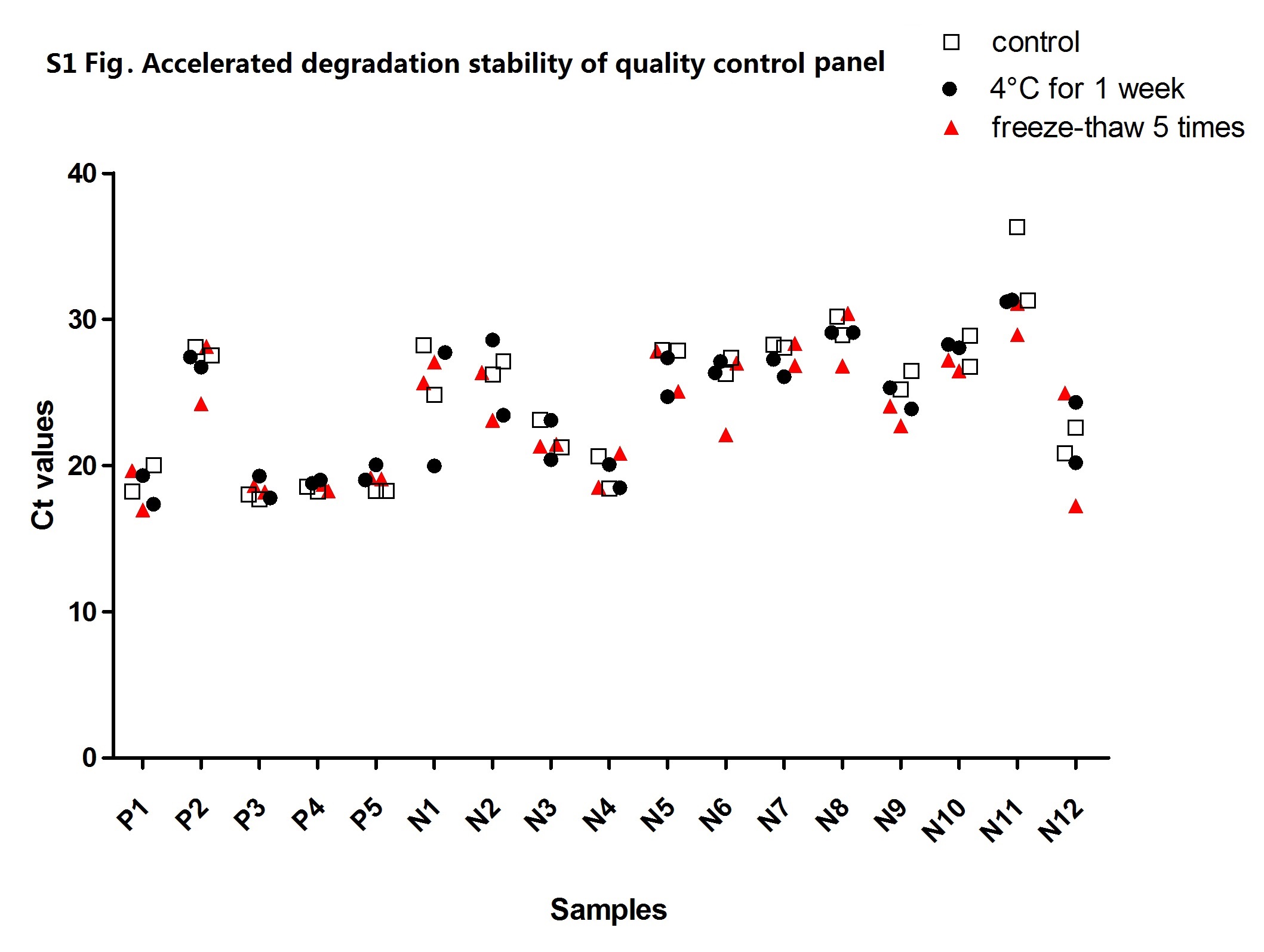

Supplement: S1 Fig — Open squares indicate results obtained for samples stored at -70°C, which were treated as controls. Filled circles indicate the results for samples stored at 4°C for 1 week. Red triangles indicate the results for samples that were repeatedly freeze-thawed five times. (JPG) [file pone.0137862.s001.jpg]

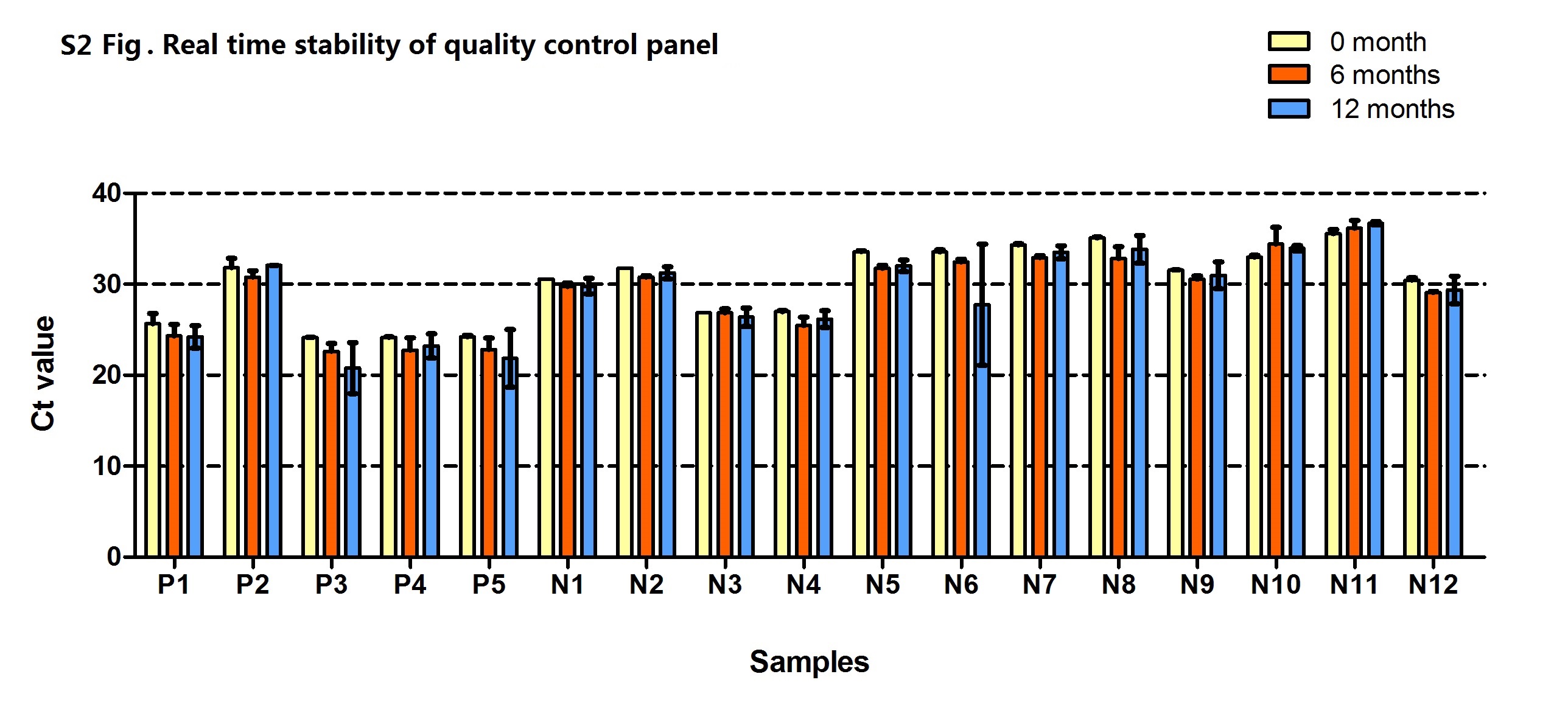

Supplement: S2 Fig — Yellow columns indicate the results for samples at the time of production, while red and blue columns indicate the results obtained for samples at 6 and 12 months after production, respectively. The Ct values of two independent tests are illustrated as the mean (column top) and standard deviation (bar). (JPG) [file pone.0137862.s002.jpg]

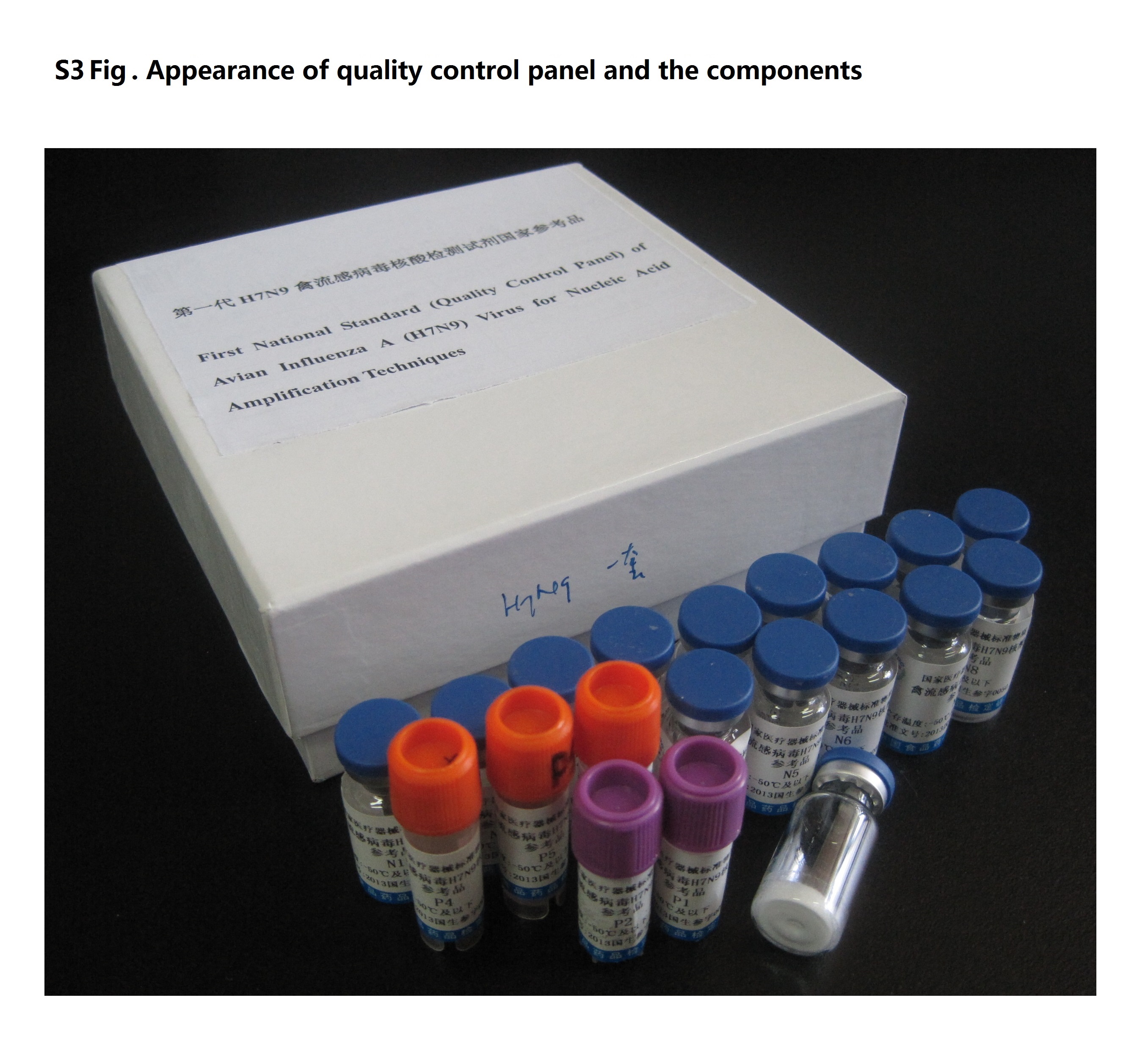

Supplement: S3 Fig — Blue-capped penicillin bottles (labeled N1-N12) contain lyophilized viral cultures of non-H7N9 influenza viruses, while vials with orange or purple caps (labeled P1-P5) contain liquid viral cultures of avian influenza A (H7N9) virus. After lyophilization, the material in the bottles appeared to be milky-white, loose, thick pie-shaped. (JPG) [file pone.0137862.s003.jpg]

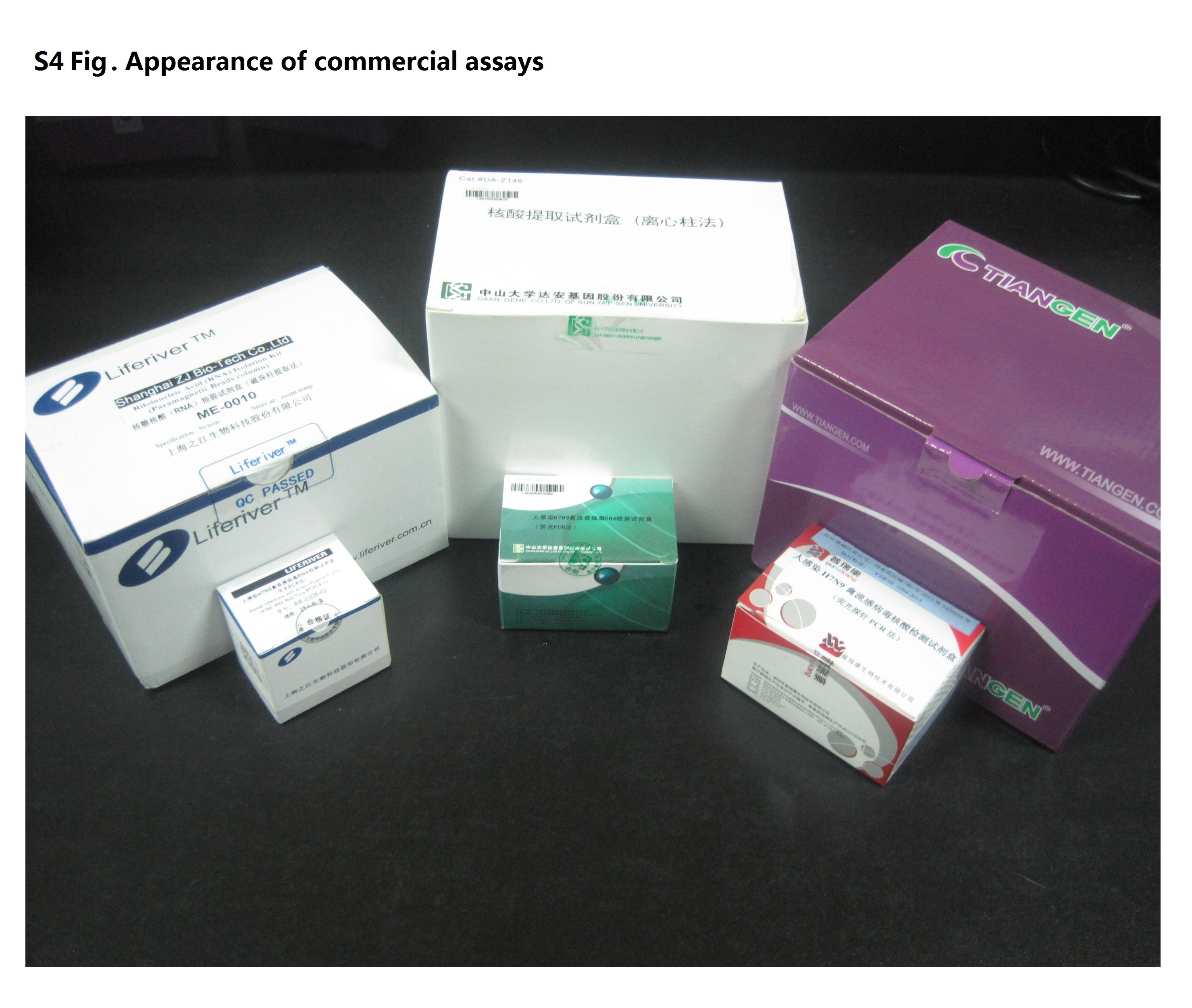

Supplement: S4 Fig — Front row from left to right: the detection kits of Liferiver, DAAN, and Puruikang. Back row from left to right: the RNA extraction reagents coupled with the detection kits of Liferiver, DAAN, and Puruikang. (JPG) [file pone.0137862.s004.jpg]
