# Supplementary material for: Evaluation of Commercial Diagnostic Assays for the Specific Detection of Avian Influenza A (H7N9) Virus RNA Using a Quality-Control Panel and Clinical Specimens in China
Source: PLoS One. 2015 Sep 11;10(9):e0137862. doi: 10.1371/journal.pone.0137862 (PMC4567293; doi:10.1371/journal.pone.0137862)
Supplement: S1 File — (DOC) [file pone.0137862.s005.doc]

**S1 File. CNIC protocol for determining influenza virus type or subtype of the samples**

The protocol and PCR primers, recommended by CNIC, Chinese CDC, are deposited on its official website (http://www.cnic.org.cn/uploadfile/2011/0810/20110810030040578.rar) as Chinese. Here, the original texts are translated into English as follows by the authors of this article.

1. RNA extraction

Reagent used in the experiment:

|  | Reagents | Catalog No. | Manufacturer |
| --- | --- | --- | --- |
| 1 | QIAGEN RNeasy Mini Kit | 74104 | Qiagen |
| 2 | β-Mercaptoethanol | M6250 | Shanghai Sangon |
| 3 | Ethanol | 10009292 | Sinopharm chemical |
| 4 | RNase-free Water | P119C | Promega |

1. Dispense 500μL RLT buffer to the 1.5mL RNase-free tubes.
2. Add the viral samples to the above tubes, mix thoroughly.
3. Add 5μL β-Mercaptoethanol to each tube, mix thoroughly and add 600μL 70% ethanol, mix thoroughly again.
4. Load 600μL mixture in step 3 to a RNeasy Mini spin column (in a 2 ml collection tube). Close the lid gently, and centrifuge for 15 s at no less than 8000 g. Discard the flow-through.
5. Carefully open the column, and repeat step 4.
6. Add 700μL Wash Buffer RW1, centrifuge for 15 s at no less than 8000 g.
7. Place the column in a new clean 2 ml collection tube and discard the flow-through. Add 500μL Wash Buffer RPE, centrifuge for 15 s at no less than 8000 g.
8. Discard the flow-through and add 500μL Wash Buffer RPE again, centrifuge for 15 s at no less than 8000 g.
9. Place the column in a clean 1.5 ml RNase-free tube. Carefully open the column and add 50 µl RNase-free Water to the center of the membrane. Incubate 3 min at room temperature.
10. Centrifuge for 1 min at not less 8000 g. Store the elution (RNA) at -70℃ until use.
11. One-step RT-PCR and sequencing

Reagent used in the experiment:

|  | Reagents | Catalog No. | Manufacturer |
| --- | --- | --- | --- |
| 1 | One-step RT PCR kit | 210212 | Qiagen |
| 2 | Primers* |  | Shanghai Sangon |
| 3 | RNase inhibitor | N2111 | Promega |
| 4 | RNase-free Water | P119C | Promega |

*: Primers are dissolved and diluted to 50μM.

The reaction cycling was listed below:

| Temperature (℃) | Time (mm:ss) | cycles |
| --- | --- | --- |
| 60 | 1:00 | 1 |
| 42 | 10:00 | 1 |
| 50 | 30:00 | 1 |
| 95 | 15:00 | 1 |
| 94 | 00:30 | 40 |
| 50 | 00:30 |
| 72 | 01:00 |
| 72 | 10:00 | 1 |
| 4 |  |  |

The reaction setup was listed below:

| Components | Volume (μL) per reaction |
| --- | --- |
| 5× QIAGEN OneStep  RT-PCR Buffer | 5 |
| 10mM dNTP Mixture | 1 |
| RT-PCR Enzyme Mix | 1 |
| RNase Inhibitor | 0.1 |
| Forward primer* | 0.5 |
| Reverse primers* | 0.5 |
| RNase Free Water | 11.9 |
| Total | 20 |

*: The primers used in the reaction were listed in Table 1 below.

Table 1. Primers used in the RT-PCR and sequencing of HA, NA and NS genes for determining influenza virus type or subtype of the samples

| Primer name | Primer sequence | Tested samples |
| --- | --- | --- |
| FluB-NS-F485* | 5’-GGG ACA TGA ACA ACA AAG ATG C-3’ | Influenza B  NS |
| FluB-NS-R988 | 5’-TGT CAG CTA TTA TGG AGC TG-3’ |
| H1HA-F768 * | 5’-ACT ACT GGA CTC TGC TGG AAC-3’ | Seasonal influenza A H1N1  and influenza A H1N1 2009pdm HA |
| H1HA-R1094 | 5’-CAA TGA AAC CGG CAA TGG CTC C-3’ |
| N1-F1059* | 5’-AAG GGG TTT TCA TAC AGG TAT GGT-3’ | Seasonal influenza A H1N1  and Influenza A H1N1 2009pdm NA |
| N1-R1165 | 5’-TCT GTC CAT CCA TTA GGA TCC-3’ |
| H3HA-F671* | 5’-ATC AGG GAG AGT CAC AGT CTC-3’ | Influenza A H3N2  HA |
| H3HA-R940 | 5’-ATG CTT CCA TTT GGA GTG ATG C-3 |
| N2-F779 * | 5’-GGA AAT CGT TCA TAT TAG CCC ATT G-3’ | Influenza A H3N2  NA |
| N2-R955 | 5’-AGC ACA CAT AAC TGG AAA CAA TGC-3’ |
| H5HA-F248 * | 5’-GTG ACG AAT TCA TCA ATG TRC CG-3’ | Influenza A H5N1  HA |
| H5HA-R647 | 5’-CTC TGG TTT AGT GTT GAT GTY CCA A-3’ |
| AN1-F580 * | 5'-TGA AGT ACA ATG GCA TAA TAA CWG ACA C-3’ | Influenza A H5N1  NA |
| AN1-R918 | 5'-CCA CTG CAT ATA TAT CCT ATT TGA TAC TCC-3’ |
| CNIC- H7F * | 5’- AGAAATGAAATGGCTCCTGTCAA-3’ | Influenza A H7N9  HA |
| CNIC- H7R | 5’- GGT TTT TTC TTG TAT TTT TAT ATG ACT TAG-3’ |
| CNIC-N9F * | 5’- TGG CAA TGA CAC ACA CTA GTC AGT-3’ | Influenza A H7N9  NA |
| CNIC-N9R | 5’- ATT ACC TGG ATA AGG GTC GTT ACA CT-3’ |

* : The primers also used as sequencing primers.
