# Supplementary material for: Evaluation of Commercial Diagnostic Assays for the Specific Detection of Avian Influenza A (H7N9) Virus RNA Using a Quality-Control Panel and Clinical Specimens in China
Source: PLoS One. 2015 Sep 11;10(9):e0137862. doi: 10.1371/journal.pone.0137862 (PMC4567293; doi:10.1371/journal.pone.0137862)
Supplement: S2 File — (DOC) [file pone.0137862.s006.doc]

**S2 File. Procedures and parameters of the freeze-drying of quality control panel.**

**1. Procedures and Parameters**

Freezing was achieved by gradual cooling to -40°C for 2 hr then holding at -40°C for 3 hr. One hundred µbar of vacuum was then applied for 0.5 hr. The actual levels of vacuum within the machine were monitored every 20~30 min and after the actual vacuum reached -30 µbar, the shelf temperature was ramped to -25°C and primary drying was continued at these settings for 15 hr. The shelf temperature was then ramped from -25 to 0°C over a period of 30 min and held at this temperature for 30min with vacuum of 100 µbar (actual vacuum -20～-25 µbar). The shelves were then ramped to 35°C within 30 min and hold for 4~6 hr before releasing the vacuum and back-ﬁlling the penicillin bottles with nitrogen. Penicillin bottles were tightly stoppered within the freeze-dryer before being removed.

**2. Typical freeze-drying curve**

µbar

℃

Hr
