# Supplementary material for: Evaluation of Commercial Diagnostic Assays for the Specific Detection of Avian Influenza A (H7N9) Virus RNA Using a Quality-Control Panel and Clinical Specimens in China
Source: PLoS One. 2015 Sep 11;10(9):e0137862. doi: 10.1371/journal.pone.0137862 (PMC4567293; doi:10.1371/journal.pone.0137862)
Supplement: S3 File — (PDF) [file pone.0137862.s007.PDF]

S3 File. Instruction of diagnostic assays detecting avian influenza A (H7N9) virus RNA.

1. DAAN assay

2. Liferiver assay

3. Puruikang assay

Instructions for Use of
Detection Kit for Avian Influenza (H7N9) Virus RNA (Fluorescence PCR)

Cat. # DA-BN472

Package: 48 tests per kit

Shelf life: 6 months

Storage and Transportation: Stable at 2-8°C during transportation, must be stored at -20±5°C upon receipt and repeat freeze-thawing <5 times.

TABLE OF CONTENTS

INTENDED USE	Page 1
PRINCIPLE	Page 2
MATERIALS PROVIDED IN THE KIT	Page 2
MATERIALS REQUIRED BUT NOT PROVIDED	Page 2
WARNINGS AND PRECAUTIONS	Page 3
SPECIMENS	Page 4
PROCEDURE	Page 4
A. RNA EXTRACTION	Page 4
B. PCR PREPARATION	Page 4
C. SETTING THE REAL-TIME PCR THERMOCYCLER	Page 4
D. RESULT ANALYSIS	Page 5
E. RESULT DETERMINATION	Page 5
PROCEDURE LIMITATIONS	Page 6
PERFORMANCE CHARACTERISTICS	Page 6
MANUFACTURER	Page 6

INTENDED USE

Flu viruses can be classified as A, B, C types. Among them, according to viral hemagglutinin (HA) gene, influenza A can be divided into 16 different subtypes. According to viral neuraminidase (NA) gene, influenza A can be divided into 9 different subtypes. Different subtypes of HA can be combined with different subtypes of NA to form different flu viruses. And the birds, especially waterfowls are the natural reservoir of all influenza viruses. Avian influenza A H7N9 virus is one of the flu virus that can infect birds and humans. Persons who are infected H7N9 avian flu appear severe pneumonia. Symptoms include fever, cough and breathing difficulty, etc.
This kit is suitable for the detection of H7N9 RNA in nasopharyngeal swab and sputum specimens. It can be used to help the laboratory diagnosis of the virus infection.

PRINCIPLE

This kit, using real time PCR technique, takes a high conservative region of genome NA and genome HA in H7N9 virus as the target region. It designs specific primers and fluorescent probes to qualitatively detect AIV H7N9 by the method of real time PCR amplification. In addition to a pair of specific primers and a specific fluorescent probe, reaction system also includes PCR reaction buffer, reverse transcriptase, Hot-Start Taq enzyme, nucleotide monomers (dNTPs), $Mg2^{+}$ and other ingredients, which can achieve a specific and sensitive detection for AIV H7N9 RNA.

MATERIALS PROVIDED IN KIT

Name		Content
PCR Detection Reagent	AIV H7 PCR reaction solution A	1 x 816 µl
	AIV N9 PCR reaction solution A	1 x 816 µl
	AIV H7N9 PCR reaction solution B	1 x 228 µl
Quality Control	Internal control	1 x 240 µl
	Negative control	1 × 200 µl
	AIV H7N9 positive control	1 × 200 µl

Please note the components from different batches cannot be used interchangeably.
The reagent kit shall be stored at -20±5℃. The shelf life is 9 months. Avoid repeated freezing and thawing.
Applicable Instruments: ABI Prism 7300, ABI Prism 7500, Roche LightCycler480.

MATERIALS REQUIRED BUT NOT PROVIDED

- Programmable fluorescence detection optical system (Real-time PCR thermocycler)
- Laminar airflow hood
- 1.5 ml centrifuge tubes
- PCR reaction tubes
- Disposable latex powder-free gloves or similar material
- Bench micro-centrifuge (3,000 – 12,000 RPM)
- Vortex
- Sterile micropipettes and tips with aerosol filter or positive displacement (0.5-10 µl, 2-20 µl, 5-50 µl, 50-200 µl)

WARNINGS AND PRECAUTIONS

This kit is exclusively designed for in vitro diagnostics use.

1. Please read instructions of use carefully prior to use.

2. Handle and dispose of all biological specimens as if they were able to transmit infectious agents. Avoid direct contact with the biological specimens. Avoid spills or aerosol.

3. Handle and dispose of all reagents and materials used to carry out the assay as if they were able to transmit infectious agents. Avoid direct contact with the reagents. Avoid producing spills or aerosol. Waste must be handled and disposed of according to adequate safety measures.

4. Molecular biology procedures, such as nucleic acid extraction, reverse transcription; amplification and detection require qualified staff to avoid the risk of erroneous results especially due to the degradation of nucleic acids contained in the samples or sample contamination by amplification products.

5. It is necessary to have available separate areas for the extraction/preparation of amplification reactions and for the amplification/detection of amplification products. Never introduce an amplification product in the area designed for extraction/preparat`ion of amplification products.

6. All samples and reagents must be handled under a laminar flow hood. The pipettes must be of the positive dispensation type or be used with aerosol filter tips. The tips employed must be sterile, free from DNases and RNases, free from DNA and RNA.

7. Thaw reagents completely before use, centrifuge at 8,000rpm for 10 seconds, but avoid repeated freeze-thawing of reagents.

8. Do not use kit after the expiry date provided.

SPECIMENS

This kit must be used with the following biological specimens: nasopharyngeal swab and sputum.

All specimens must be collected in accordance to laboratory guidelines. Specimens should be transported at 0-8°C and can be stored at -20°C for a period of six months.

PROCEDURE

A. RNA Extraction

It is recommended to carry out RNA extraction on 200 µl fluid sample. Nucleic acid extraction kit from DAAN GENE CO., LTD. OF SUN YAT-SEN UNIVERSITY can be chosen for this purpose. Please follow the Instructions for Use of the kit. Other appropriate commercial kits are also optional.

The internal control provided by this kit should be extracted in coupled with samples according to the procedure described in the instruction of nucleic acid extraction kit. In common, 4µl internal control is added into 200µl samples during the procedure of RNA extraction.

Negative control in the kit should be subjected to the extraction process, but AIV H7N9 positive control should not. Negative control is for environment monitoring and positive control is for the quality control of amplification efficiency of PCR detection reagents.

B. PCR Preparation

1. Take several PCR reaction tubes. Add 17 µl of AIV H7 PCR reaction solution A (provided in the kit) and 3 µl of AIV H7N9 PCR reaction solution B (provided in the kit) to every tube.
2. Take another several PCR reaction tubes. Add 17 µl of AIV N9 PCR reaction solution A (provided in the kit) and 3 µl of AIV H7N9 PCR reaction solution B (provided in the kit) to every tube.
3. Add 5 µl of samples prepared from Section A. RNA Extraction (including negative control, positive control, and clinical samples) to the PCR reaction tubes prepared in steps 1 and 2.
4. Centrifuge at 8,000 rpm for 30 seconds.
5. Load tubes into the real-time PCR thermocycler and proceed to section C. Setting the Real-time PCR thermocycler.

C. Setting the Real-Time PCR Thermocycler

Please note procedure is for ABI7500, for all other Real-time PCR thermocycler please refer to manufacturer's operation manual.

1. Open the ABI7500 software.
2. Set "reporter dye1" as FAM, "reporter dye2" as VIC.
3. Set "quencher dye1" and "quencher dye2" as NONE.
6. Set "passive reference" as NONE.
7. Set the type of reaction as negative control (NTC), sample (UNKNOWN) or positive control according to the position of the tube in the instrument wells.
8. Set the cycling conditions:

Program	Temperature	Time (min)	Cycles
1	50°C	15:00	1
2	95°C	15:00	1
3	94°C<br>55°C	0:15<br>0:45	45

9. Set “Data Collection” at “stage 3, step 2 (55°C@0:45)”.
10. Save and click run.

D. Result Analysis

1. Click “Results” and select “Amplification Plot”.
2. Click “Graph Settings” and select “Linear”.
3. Set threshold, according to amplification curve.
4. Set baseline according to the amplification curve or start value (between 3 and 15) and end value (between 5 and 20).
5. Adjust threshold line above the amplification curve of NC samples.
6. Click “Analyze”.

E. Result Determination

1. Quality Control

Quality Control	H7/N9 FAM Amplification Curve	H7/N9 FAM	H7/N9 VIC	Result
Negative Control	No increased logarithmic phase	Undet or No Ct	Amplification curve observed	VALID
Positive Control	Obvious increase in logarithmic phase	≤ 32	Undet or No Ct	VALID

Please note if the above quality control requirements are not met, the assay is considered invalid and must be repeated.

2. Specimen

FAM Ct	FAM Amplification Curve	VIC Amplification Curve	Result
> 42	No increased logarithmic phase	Obvious increase in logarithmic phase	Negative
≤ 42	Obvious increase in logarithmic phase	increased OR No increased logarithmic phase	Positive

1) If both the amplification curves of AIV H7 and N9 meet the requirements of sentence to negative, test result is negative.

2) If both the amplification curves of AIV H7 and N9 meet the requirements of sentence to positive, test result is positive.

3) If just one of the amplification curves of AIV H7 or N9 meets the requirements of sentence to positive, testing should be repeated. If the repeated test results for the H7 and N9 are both positive, it is sentenced to H7N9 avian flu virus positive. But if the repeat test results for the H7 (or N9) is just single positive, it is sentenced to H7 avian influenza virus subtype (or N9) positive. And other methods are recommended for further confirmation

PROCEDURE LIMITATIONS

Cross-contamination occurring during specimen collection, processing, transportation and storage will result in false results. Gene mutations of virus in the process of spread will result in a false negative result. Due to the extraction principle, the extraction efficiency varies in different extraction kits. And the Nucleic acid extraction kit (Spin Columns Method) from DAAN GENE CO., LTD. OF SUN YAT-SEN UNIVERSITY is recommended.

PERFORMANCE CHARACTERISTICS

Sensitivity: 1.0×10^3 copies per ml

Other viruses with similar infection site or infectious symptoms (such as influenza A virus H3, influenza A virus H1, influenza B virus, parainfluenza virus, avian influenza virus H5, avian influenza virus H9) have no cross-reaction.

MANUFACTURER

DAAN GENE CO., LTD. OF SUN YAT-SEN UNIVERSITY
Manufacturing Address: 19 Xiangshan Road, Science Park, High & New Technology Development District, Guangzhou, Guangdong, P. R. China
Post code: 510665
Tel: +86-20-32290789, Fax: +86-20-32068126 Website: http://www.daangene.com

North America distributor Address: 200 - 5050 Kingsway, Burnaby, B.C. Canada
Post code: V5H 4H2
Tel: 604 451 7588, Fax: 604 451 7587, Toll Free: 1 877 651 7588

Ordering and consulting Email address: order@daandiagnostics.com, info@daandiagnostics.com

Liferiver™

Revision No.: ZJ0003

Issue Date: Jun 3rd, 2013

Avian Influenza Virus H7N9 Real Time RT-PCR Kit User Manual

For In Vitro Diagnostic Use Only

Σ 25 -20 °C IVD

REF **RR-0309-02**

For use with ABI Prism®7000/7300/7500/7900/Step One Plus; iCycler iQ™4/iQ™5; Smart Cycler II;Bio-Rad CFX 96;Rotor Gene™6000; Mx3000P/3005P;MJ-Option2/Chromo4; LightCycler®480 Instrument

Shanghai ZJ Bio-Tech Co., Ltd.
www.liferiver.com.cn Tel: +86-21-34680596
trade@liferiver.com.cn Fax: +86-21-34680595
2nd floor,No.15 Building,No.188 Xinjunhuan road,
PuJiang Hi-tech Park Shanghai China

1. Intended Use

Avian influenza virus H7N9 real time RT-PCR kit is used for the detection of gene H7 and gene N9 of avian influenza A subtype H7N9 in human nasal and pharyngeal secretions and bird fece by using real time PCR systems.

2. Principle of Real-Time PCR

The principle of the real-time detection is based on the fluorogenic 5'nuclease assay. During the PCR reaction, the DNA polymerase cleaves the probe at the 5' end and separates the reporter dye from the quencher dye only when the probe hybridizes to the target DNA. This cleavage results in the fluorescent signal generated by the cleaved reporter dye, which is monitored real-time by the PCR detection system. The PCR cycle at which an increase in the fluorescence signal is detected initially (Ct) is proportional to the amount of the specific PCR product. Monitoring the fluorescence intensities during Real Time allows the detection of the accumulating product without having to re-open the reaction tube after the amplification.

3. Product Description

Highly pathogenic avian influenza (HPAI) caused by certain subtypes of influenza A virus in animal populations, particularly chickens, poses a continuing global human public health risk. Direct human infection by an avian influenza A (H5N1) virus was first recognized during the 1997 outbreak in Hong Kong. The avian influenza virus H7N9 is one subgroup among the larger group of H7 viruses. Some cases of human infection with H7N9 virus in China are confirmed till early April of 2013.

Avian influenza virus H7N9 real time RT-PCR kit contains a specific ready-to-use system for the detection of avian influenza virus H7N9 by Reverse Transcription Polymerase Chain Reaction (RT-PCR) in the real-time PCR system. The master contains Super Mix for the specific amplification of the avian influenza virus H7N9 RNA. The reaction is done in one step real time RT-PCR. The first step is a reverse transcription (RT), during which the avian influenza virus H9 RNA is transcribed into cDNA. Afterwards, a thermostable DNA polymerase is used to amplify the specific gene fragments by polymerase chain reaction. Fluorescence is emitted and measured by the real time systems´ optical unit during the PCR. The detection of amplified avian influenza virus H7N9 DNA fragment is performed in fluorimeter **channel** FAM and **HEX/VIC/JOE** with the fluorescent quencher BHQ1. In addition, the kit contains a system to identify possible PCR inhibition by measuring the Cal Red 610/ROX/TEXAS RED fluorescence of the internal control (IC).

4. Kit Contents

Ref.	Type of reagent	Presentation 25rxns
1	H7N9 Super Mix	1 vial, 480µl
2	RT-PCR Enzyme Mix	1 vial, 28µl
3	Molecular Grade Water	1 vial, 400µl
4	H7N9 Internal Control	1 vial, 30µl
5	H7N9 Positive Control	1 vial, 30µl

Analysis sensitivity: 1×10³copies/ml;

Note: Analysis sensitivity depends on the sample volume, elution volume, nucleic acid extraction methods and other factors .If you use the RNA extraction kits recommended, the analysis sensitivity is the same as it declares. However, when the sample volume is dozens or even hundreds of times greater than elution volume by some concentrating method, it can be much.

5. Storage

- All reagents should be stored at -20°C. Storage at +4°C is not recommended.
- All reagents can be used until the expiration date indicated on the kit label.
- Repeated thawing and freezing (> 3x) should be avoided, as this may reduce the sensitivity of the assay.
- Cool all reagents during the working steps.
- Super Mix should be stored in the dark.

6. Additionally Required Materials and Devices

- Biological cabinet
- Vortex mixer
- Cryo-container
- Sterile filter tips for micro pipets
- Disposable gloves, powderless
- Refrigerator and Freezer
- Real time PCR system
- Real time PCR reaction tubes/plates
- Pipets (0.5µl – 1000µl)
- Sterile microtubes
- Biohazard waste container
- Tube racks
- Desktop microcentrifuge for "eppendorf" type tubes (RCF max. 16,000 x g)

7. ⚠Warnings and Precaution

Carefully read this instruction before starting the procedure.

- For in vitro diagnostic use only.
- This assay needs to be carried out by skilled personnel.
- Clinical samples should be regarded as potentially infectious materials and should be prepared in a laminar flow hood.
- This assay needs to be run according to Good Laboratory Practice.
- Do not use the kit after its expiration date.
- Avoid repeated thawing and freezing of the reagents, this may reduce the sensitivity of the test.
- Once the reagents have been thawed, vortex and centrifuge briefly the tubes before use.
- Prepare quickly the Reaction mix on ice or in the cooling block.
- Set up two separate working areas: 1) Isolation of the RNA/ DNA and 2) Amplification/ detection of amplification products.
- Pipets, vials and other working materials should not circulate among working units.
- Use always sterile pipette tips with filters.
- Wear separate coats and gloves in each area.
- Do not pipette by mouth. Do not eat, drink, smoke in laboratory.
- Avoid aerosols

8. Sample Collection, Storage and transport

- Collected samples in sterile tubes;
- Specimens can be extracted immediately or frozen at -20°C to -80°C.
- Transportation of clinical specimens must comply with local regulations for the transport of etiologic agents

9. Procedure

9.1 RNA-Extraction

Different brand RNA extraction kits are available. You may use your own extraction systems or the commercial kit based on the yield. For the RNA extraction, please comply with the manufacturer's instructions. The recommended Extraction kit is as follows:

Nucleic Acid Isolation Kit	Cat. Number	Manufacturer
RNA Isolation Kit	ME-0010/ME-0012	ZJ Biotech
QIAamp Viral RNA Mini extraction Kit (50)	52904	QIAGEN

9.2 Internal Control

It is necessary to add internal control (IC) into the extraction lysis buffer. Internal Control (IC) allows the user to determine and control the possibility of PCR inhibition. Add the internal control (IC) 1µl/samples and the result will be shown in the Cal Red 610/ROX/TEXAS RED.

9.3 RT-PCR Protocol

The Master Mix volume for each reaction should be pipetted as follows:

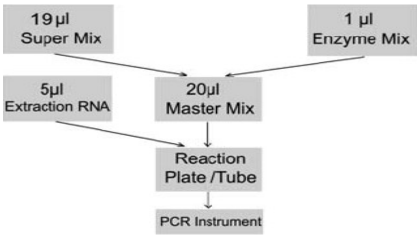

※PCR system without Cal Red 610/ROX/TEXAS RED channel may be treated with 1µl Molecular Grade Water instead of 1µl IC.

1) The volumes of Super Mix and Enzyme Mix per reaction multiply with the number of samples, which includes the number of controls, standards, and sample prepared. Molecular Grade Water is used as the negative control. For reasons of unprecise pipetting, always add an extra virtual sample. Mix completely then spin down briefly in a centrifuge.

2) Pipet **20µl** Master Mix with micropipets of sterile filter tips to each of the *Real time* PCR reaction plate/tubes. Separately add **5µl** RNA sample, positive and negative controls to different reaction plate/tubes. Immediately close the plate/tubes to avoid contamination.

3) Spin down briefly in order to collect the Master Mix in the bottom of the reaction tubes.

4) Perform the following protocol in the instrument:

45°C for 10min	1cycle
95°C for 15min	1cycle
95°C for 15sec, 60°C for 1min (Fluorescence measured at 60°C)	45cycles

Selection of fluorescence channels	
FAM	H7
HEX/VIC/JOE	N9
Cal Red 610/ROX/TEXAS RED	IC

5）⚠If you use ABI Prism® system, please choose **"none"** as **passive reference** and **quencher.**

10. Threshold setting: just above the maximum level of molecular grade water.

11. Quality control: Negative control, internal control and positive control must be performed correctly, otherwise the sample results are invalid.

Channel / Control	Ct value		
	FAM	HEX/VIC/JOE	Cal Red 610/ROX/ TEXAS RED
Molecular Grade Water	UNDET	UNDET	≤43
Positive Control	≤35	≤35	——

12. Data Analysis and Interpretation： The following results are possible:

	Ct value			Result Analysis
	FAM	HEX	Cal Red 610	
1#	UNDET	UNDET	≤43	Below the detection limit or negative
2#	≤43	UNDET	——	Gene H7 positive;
3#	UNDET	≤43	——	Gene N9 positive;
4#	≤43	≤43	——	H7N9 Positive;
5#	43～45		≤43	Re-test; If it is still 43~45, report as 1#
6#	UNDET		UNDET	PCR Inhibition; No diagnosis can be concluded.

For further questions or problems，please contact our technical support at **trade@liferiver.com.cn**

【Production Name】

Detection Kit for human infection with avian influenza virus（H7N9） RNA (Fluorescence PCR)

【Package Size】

24 Test/kit

【Intended Use】

Human infection with avian influenza virus (H7N9) that belongs to influenza A virus, Orthomyxoviridae, has not been reported before 2013. The particle of this novel virus is polygon-shaped and with envelops, of which genome is a segmented, single-strand sense RNA.

Avian influenza virus (H7N9) is transmitted by air-bone, close contact with secretion and feces of infected poultry, and direct virus contact. So far, there is no solid evidence of human-human transmission, however, the virus tends to infect human easier because the mutations of the virus were found. The confirmed cases were all adults, and the high-risk population was mainly persons whose job is raising, butchering, manufacturing and selling of poultry and who contacted poultry one week before illness. The common symptoms are flu-like symptom, e.g., fever, cough, scanty sputum, sometimes with headache, muscle ache and body discomfort. The disease of severe cases will progress rapidly and become severe pneumonia, with body temperature maintains above 39℃, dyspnea, with or without sputum of hemoptysis; or rapidly progress to be acute respiratory distress syndrome (ARDS), mediastinal emphysema, sepsis, stroke, conscious disturbance, and acute kidney injury et. al. Therefore, the early diagnostics, discovery and treatment of human infection with avian influenza virus (H7N9) are critical important.

This kit, based on a complex real time PCR technique, is used to qualitatively detect the avian influenza virus (H7N9) RNA of the nasopharyngeal/throat swab specimens from patients with influenza-like syndrome or related close contactors. The detection results can be referred to help diagnose the virus infection, but can not be regarded as the sole diagnostic evidence.

【Principle of the Detection】

This detection kit is based on the real time fluorescent PCR technique. The targets are the conserved regions of HA and NA genes of avian influenza virus (H7N9). RNA of avian influenza virus (H7N9) of nasopharyngeal/throat swab specimens is detected qualitatively through realtime fluorescent PCR, including H7-specific primers and probes, N9-specific primers and probes, PCR mixture, reverse transcription enzyme and DNA polymerase.

【Kit Components】

Reagent type	Component Name	Size	Contained Material
PCR Reaction Reagents	PCR mix A-H7	451.2μl×1 tube	H7 primer and probe, IC primer and probe, 2×RT-PCR buffer
	PCR mix B-N9	451.2μl×1 tube	N9 primer and probe, IC primer and probe, 2×RT-PCR buffer
	PCR mix C	28.8μl×1 tube	DNA pol、RT enzyme
	PCR mix D	28.8μl×1 tube	DNA pol、RT enzyme
Controls	Negative Control	400μl×1 tube	Cell culture medium
	Positive Control -H7	400μl×1 tube	Bacteriophage containing H7 target region
	Positive Control -N9	400μl×1 tube	Bacteriophage containing N9 target region

Notes：Components of different lot kit can not be exchanging-used；Internal Control (IC) is designed to detect the housekeeping gene of human cells。

【Storage and Expiry】

All reagents should be stored at -20°C and protected from light.

Repeated thawing and freezing should be avoided (no more than 4 times).

Expire is 6 months, and the opened reagents are stable at 4℃ for 15 days.

【Required Equipment】

Dual channel fluorescent PCR machine, includes Bio-Rad CFX96, ABI 7500, Roche Light Cycler 480.

【Specimens Requirement】

1. Specimen Type：Nasopharyngeal/throat swab
2. Specimen Collection

Bilateral tonsils and posterior pharyngeal wall are swabbed gently with force using swabs with polypropylene fiber head, but the tongue should be avoided to be touched. Place the swab in the transport tube and roll the swab 5 times in 1ml collection liquid of the transport tube. Discard the tail of the swab and screw the cap tightly.

3. Storage and transport

Specimens can be stored at 4℃ for 48h，and should be stored at -70℃ or below if more than 48h. Repeat freeze-thaw should be avoided (no more than 3 times).

Specimens should be transported by professional personnel; Ice bags should be used for short-term transportation but dry ice should be used for long-term transportation.

【Procedure】

1. RNA Extraction (In Sample Preparation Area)

1.1 The viral nucleic acid extraction kit (spin-column method) produced by Shenzhen Puruikang Co. Ltd. (Cat No.: S070001) or kit produced by TIANGEN BIOTECH (BEIJING), Co., Ltd (Cat No.: DP315-R) are recommended to be used. Generally, 200μl specimen is needed for RNA extraction that is conducted according to the instruction of nucleic acid extraction kit.

1.2 The negative and positive controls contained in the detection kit are extracted in along with specimens.

2. PCR Mix Preparation (In Reagent Preparation Area)

2.1 PCR reaction mix is prepared as follows (n=reaction number):

H7 PCR reaction:

PCR mix A-H7 18.8μl × n, PCR mix C 1.2 μl × n.

N9 PCR reaction:

PCR mix B- N9 18.8μl × n, PCR mix D 1.2 μl × n.

(CAUTIONS：Make SURE PCR MIX A/B THAW COMPLETELY AND PCR MIX C/D CENTRIFUGED TO THE BOTTOM BEFORE USE.)

2.2 Aliquot 20μl PCR reaction mix to each PCR reaction tub, then move to Sample Preparation Area.

3. Sample Adding (In Sample Preparation Area)

Pipette 10μl of extracted specimens, negative control and positive control each by tips with aerosol filter to PCR reaction tube with reaction mix. Cap the tube, mix gently, spin several seconds, and then move to PCR Amplification Area.

4. PCR Amplification and Detection (PCR Amplification Area)

4.1 Place the PCR reaction tubes on the PCR machine and cover the lid.

4.2 Setup of cycling parameter (Bio-Rad CFX96，ABI 7500)

Program	Cycles	Temperature Target(℃)	Hold Time (min:sec)	Acquisition Mode
1	1	45	30:00	None
2	1	90	0:30	None
3	40	95	0:10	None
		55	0:30	Single
4	1	25	0:10	None

Setup of cycling parameter (Roche Light Cycler 480)

Program	Cycles	Temperature Target(℃)	Hold Time (min:sec)	Acquisition Mode
1	1	45	30:00	None
2	1	90	0:30	None
3	40	93	0:10	None
		55	0:30	Single
4	1	37	0:10	None

4.3 Setting of detection channel：FAM for H7 and N9，HEX/VIC for IC.

5. Quality Control Requirement

5.1 Negative Control (NC)：Negative result and Ct value of IC＜33。

5.2 Positive Control (PC)：Positive result and Ct value≤33。

5.3 The criteria of 5.1 and 5.2 should be both meet for one test, otherwise the test is invalid and should be repeated.

6. Data Analysis

Set the baseline to be 6 to 15 cycle (FAM) and 10 to 20 cycle (HEX/VIC), then adjust the threshold line above the signal curve of NC (noise signal

curve).

【Reference Range】

Negative: Ct value=40 or no value ('undetected');

Positive: Ct value≦37;

Borderline: 40＞Ct value＞37

【Interpretation of results】

1. NC, PC of the kit should be tested in parallel with samples within a run, otherwise the test is invalid.
2. Interpretation

2.1 Negative result (-): amplification curve is not observed in FAM channel but observed in HEX/VIC channel (IC Ct＜33); if amplification curve in HEX/VIC channel is not observed or IC Ct≥33, the test should be repeated.

2.2 Positive result (+): amplification curve is observed in FAM channel and Ct ≤37.

2.3 Borderline: amplification curve is observed in FAM channel and 40＞Ct >37, the test should be repeated.

2.4 Repeat test mentioned in 2.1 and 2.3 is performed as below:

Sample is recommended to be concentrated according to the procedure: centrifuge the collection liquid of the transport tube at 12000rpm, 4℃for 5min. Discard supernatant carefully and leave about 200μl remaining at the bottom of the tube. Extract the remaining according to standard protocol. If amplification curve observed, the result of retest is positive, otherwise it is negative.

2.5 When control criteria are meet, the interpretation modes for a clinical sample are shown as follows:

PCR reaction	Mode1	Mode 2	Mode 3	Mode 4
H7	+	+	-	-
N9	+	-	+	-
Interpretation	avian influenza virus (H7N9) RNA positive	Only H7 subtype influenza virus RNA positive	Only N9 subtype influenza virus RNA positive	avian influenza virus (H7N9) RNA negative

PS: IC of the sample with negative result should be positive and Ct value＜33; IC of the sample with positive result is not required.

3. Demonstration of results interpretation

Two clinical samples were detected on ABI7500 PCR machine and the result was shown as Figure1and Figure 2.

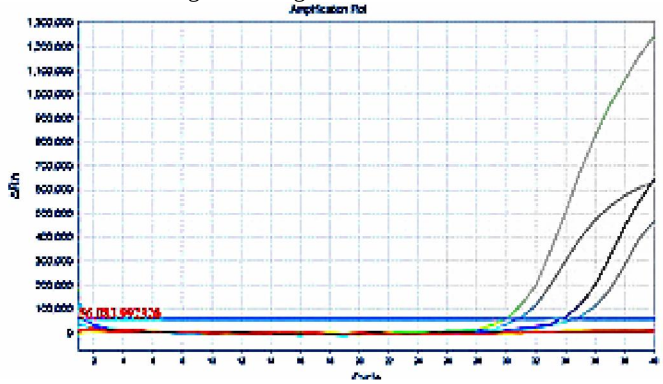

Figure 1. Amplification curve

E6	H7 Target 1	UNKNOWN	FAM-TAM...	30.090
E6	H7 Target 2	UNKNOWN	VIC-TAMRA	31.371
F7	N9 Target 1	UNKNOWN	FAM-TAM...	30.909
F7	N9 Target 2	UNKNOWN	VIC-TAMRA	31.263
D9	H7 Target 1	UNKNOWN	FAM-TAM...	32.685
D9	H7 Target 2	UNKNOWN	VIC-TAMRA	31.733
F4	N9 Target 1	UNKNOWN	FAM-TAM...	32.983
F4	N9 Target 2	UNKNOWN	VIC-TAMRA	31.226
C7	H7 Target 1	UNKNOWN	FAM-TAM...	Undeter...
C7	H7 Target 2	UNKNOWN	VIC-TAMRA	29.059
H8	N9 Target 1	UNKNOWN	FAM-TAM...	Undeter...
H8	N9 Target 2	UNKNOWN	VIC-TAMRA	29.710
H7	H7 Target 1	UNKNOWN	FAM-TAM...	Undeter...
H7	H7 Target 2	UNKNOWN	VIC-TAMRA	29.949
C8	N9 Target 1	UNKNOWN	FAM-TAM...	Undeter...
C8	N9 Target 2	UNKNOWN	VIC-TAMRA	27.643

Figure 2. Ct value

As illustrated in the above figures: Ct value of PC-H7 is 30.090, Ct value of PC-N9 is 30.909; the result of NC is negative and IC Ct value of H7 is 29.059, IC Ct value of N9 is 29.710, which meet the quality control criteria. Therefore the result of test is valid and can be reported as:

Positive result can be reported as: avian influenza virus (H7N9) RNA is detectable in the sample with concentration higher than LoD of kit;

Negative result can be reported as: avian influenza virus (H7N9) RNA is undetectable in the sample with concentration lower than LoD of kit

【Limitations】

1. The results of this kit are referred for the clinical diagnosis, but can not be regarded as the only diagnostic evidence.
2. The false negative results could be resulted from the follow reasons:

2.1 Improper sample collection, transportation or extreme low viral load in the specimens;

2.2 The detection sequence of avian influenza virus (H7N9) is changed naturally or by other reasons such as antiviral treatment.

3. Cross-containment by positive samples would lead to false positive results.

【Notes】

1. For in vitro diagnostic use only, please carefully read the content of this instruction manual before use.
2. Clinical samples should be regarded as potentially infectious materials and should be prepared in a laminar flow hood.
3. The positive control of kit is in vitro transcribed RNA, and the negative control is saline water, which are non-infectious. However, the above components should be handled as carefully as infectious materials.
4. The laboratory operators should be trained for the techniques of gene amplification or molecular detection methods, and be certified for related testing operation. The laboratory should owe suitable bio-safe protection facilities and protocols and run according to the requirement of molecular diagnosis, clinical gene amplification laboratory. The work laboratory should have separate rooms including reagent preparation area, specimen preparation area and amplification/detection area. The materials of each area can not be exchange-used and the work flow of persons and air should be required strictly to avoid cross-containment. Experiment materials (Eppendorf tube, tips and et. al) should be clean properly with validation procedure to avoid the false negative results caused by RNase or PCR inhibitor containment.
5. All components of kit should be thawed thoroughly with shaking before use, but repeated freeze-thaw should be prevented. Centrifuge the reagent tubes for several seconds before open.
6. RNA samples is recommended to be tested immediately, otherwise please store the samples at -70℃ for no more than one week。
7. Avoid foams when dispensing PCR mix as much as possible. Check the cap of PCR reaction tube to be fit before loaded to machine.
8. Clean the bench and pipettes with 500-2000ppm sodium hypochlorite or 75% ethanol or UV-ray after experiments.

【Contact】

Company Name: Shenzhen Puruikang Biotech Co., Ltd

Company Address: Research Center, #9 TAOHUAYUAN Technique Innovation Park, Xixiang, Baoan District, Shenzhen city, Guangdong.

Manufacture Address：Building 1, 2, HAOYE Industry Park, Xixiang, Baoan District, Shenzhen city, Guangdong; Research Center, #9 TAOHUAYUAN Technique Innovation Park, Xixiang, Baoan District, Shenzhen city, Guangdong.

Post Code：518102

Tel : 0755-29196898

Fax: 0755-29190918

Webpage：http://www.szprk.com
