# Supplementary material for: Evaluation of Commercial Diagnostic Assays for the Specific Detection of Avian Influenza A (H7N9) Virus RNA Using a Quality-Control Panel and Clinical Specimens in China
Source: PLoS One. 2015 Sep 11;10(9):e0137862. doi: 10.1371/journal.pone.0137862 (PMC4567293; doi:10.1371/journal.pone.0137862)
Supplement: S1 Table — (DOC) [file pone.0137862.s008.doc]

S1 Table. Influenza virus strains and their final concentrations in the reference panel.

| Vial | The name of virus strains | Type and subtype of the influenza virus | Concentration of each vial of final panel | | Storage type | The sites for virus culturing |
| --- | --- | --- | --- | --- | --- | --- |
|  |  |  | Virus titer | RNA Log_10_ copies/µl |  |  |
| P1 | A/Zhejiang/DTID-ZJU01/2013 | Influenza A H7N9 | 10^5.7^ TCID50/mL | 7.83 | Liquid | State Key Laboratory for Diagnosis and Treatment of Infectious Diseases, Zhejiang University, China |
| P2 | A/Zhejiang/DTID-ZJU02/2013 | Influenza A H7N9 | 10^4.6^  TCID50/mL | 8.88 |  |  |
| P3 | A/Shanghai/1/2013 | Influenza A H7N9 | 0.64 HAU | 6.93 |  | Chinese National Influenza Center, National Institute for Viral Disease Control and Prevention, Chinese Center for Disease Control and Prevention, China |
| P4 | A/Shanghai/2/2013 | Influenza A H7N9 | 0.32 HAU | 6.78 |  |  |
| P5 | A/Anhui/1/2013 | Influenza A H7N9 | 0.32 HAU | 6.63 |  |  |
| N1 | B/Guangdongluohu/15/2007 | Influenza B | 0.13 HAU | 5.58 | Freeze-dried |  |
| N2 | B/Fujianxinluo/54/2006 | Influenza B | 0.13 HAU | 5.47 |  |  |
| N3 | B/Tianjin/2/2001 | Influenza B | 0.13 HAU | 6.66 |  |  |
| N4 | A/Hiroshima/52/2005 | Influenza A H3N2 | 0.19 HAU | 5.15 |  |  |
| N5 | A/Hanfang/359/1995 | Influenza A H3N2 | 0.05 HAU | 4.78 |  |  |
| N6 | A/Wisconsin/67/2005 | Influenza A H3N2 | 0.32 HAU | 5.00 |  |  |
| N7 | A/Guangdongluohu/219/2006 | Influenza A Seasonal H1N1 before 2009 | 0.13 HAU | 4.79 |  |  |
| N8 | A/Hufang/7/1999 | Influenza A Seasonal H1N1 before 2009 | 0.16 HAU | 4.30 |  |  |
| N9 | A/Beijing/SWL5/2009 | Influenza A H1N1 pdm09 | 1.28 HAU | 5.49 |  |  |
| N10 | A/Califonia/07/2009 | Influenza A H1N1 pdm09 | 0.13 HAU | 4.31 |  |  |
| N11 | A/Sichuan/1/2006 | Influenza A H5N1 | 1.28 HAU | 3.61 |  |  |
| N12 | A/Xinjiang/1/2006 | Influenza A H5N1 | NA | 4.43 |  |  |

NA: not avaibable

HAU: HA Unit, HAUs listed in this table were calculated as the original virus titer divided by dilution factors.
