# Supplementary material for: Evaluation of Commercial Diagnostic Assays for the Specific Detection of Avian Influenza A (H7N9) Virus RNA Using a Quality-Control Panel and Clinical Specimens in China
Source: PLoS One. 2015 Sep 11;10(9):e0137862. doi: 10.1371/journal.pone.0137862 (PMC4567293; doi:10.1371/journal.pone.0137862)
Supplement: S2 Table — (DOC) [file pone.0137862.s009.doc]

S2 Table. Primers used for the adding of T7 sequence by PCR amplification.

| Primer name | Primer sequence (5’ to 3’) | Amplification Target |
| --- | --- | --- |
| H7-F trans | ATTGTAATACGACTCACTATAGGGATGAACACTCAAATCCTGGT | HA gene of avain influenza A virus (H7N9) |
| H7/Anhui/1/2013-R | TTATATACAAATAGTGCACC |
| H1N1 M T7trans-F | ATTGTAATACGACTCACTATAGGGATGAGTCTTCTAACCGAGGT | MP gene of influenza A virus |
| H1N1 M T7trans-R | TTACTCTAGCTCTATGTTGA |
| B-F trans | ATTGTAATACGACTCACTATAGGGGTTGCCACTGATGATCTTAC | NS gene of influenza B virus |
| B-R trans | TTTTAAATTCACAAGCACTGCCT |

The characters of sequence with underline indicate the added sequence of T7 promoter.
